# Multi-step screening of DNA/lipid nanoparticles and co-delivery with siRNA to enhance and prolong gene expression

**DOI:** 10.1038/s41467-022-31993-y

**Published:** 2022-07-25

**Authors:** Yining Zhu, Ruochen Shen, Ivan Vuong, Rebekah A. Reynolds, Melanie J. Shears, Zhi-Cheng Yao, Yizong Hu, Won June Cho, Jiayuan Kong, Sashank K. Reddy, Sean C. Murphy, Hai-Quan Mao

**Affiliations:** 1 Department of Biomedical Engineering, Johns Hopkins University School of Medicine, Baltimore, MD USA; 2 Institute for NanoBioTechnology, Johns Hopkins University, Baltimore, MD USA; 3 Translational Tissue Engineering Center, Johns Hopkins University School of Medicine, Baltimore, MD USA; 4 Department of Laboratory Medicine and Pathology, University of Washington, Seattle, WA USA; 5 Center for Emerging and Re-emerging Infectious Diseases, University of Washington, Seattle, WA USA; 6 Department of Materials Science and Engineering, Johns Hopkins University, Baltimore, MD USA; 7 Department of Chemical and Biomolecular Engineering, Johns Hopkins University, Baltimore, MD USA; 8 Department of Plastic and Reconstructive Surgery, Johns Hopkins University School of Medicine, Baltimore, MD USA; 9 Department of Microbiology, University of Washington, Seattle, WA USA; 10 Seattle Malaria Clinical Trials Center, Fred Hutch Cancer Research Center, Seattle, WA USA

**Keywords:** Transfection, DNA and RNA

## Abstract

Lipid nanoparticles hold great potential as an effective non-viral vector for nucleic acid-based gene therapy. Plasmid DNA delivery can result in extended transgene expression compared to mRNA-based technologies, yet there is a lack of systematic investigation into lipid nanoparticle compositions for plasmid DNA delivery. Here, we report a multi-step screening platform to identify optimized plasmid DNA lipid nanoparticles for liver-targeted transgene expression. To achieve this, we analyze the role of different helper lipids and component ratios in plasmid DNA lipid nanoparticle-mediated gene delivery in vitro and in vivo. Compared to mRNA LNPs and in vivo-jetPEI/DNA nanoparticles, the identified plasmid DNA lipid nanoparticles successfully deliver transgenes and mediate prolonged expression in the liver following intravenous administration in mice. By addressing different physiological barriers in a stepwise manner, this screening platform can efficiently down select effective lipid nanoparticle candidates from a lipid nanoparticle library of over 1000 formulations. In addition, we substantially extend the duration of plasmid DNA nanoparticle-mediated transgene expression using a DNA/siRNA co-delivery approach that targets transcription factors regulating inflammatory response pathways. This lipid nanoparticle-based co-delivery strategy further highlights the unique advantages of an extended transgene expression profile using plasmid DNA delivery and offers new opportunities for DNA-based gene medicine applications.

## Abstract (web summary)

Plasmid DNA offers extended transgene expression duration compared to mRNA technologies. Here, using a multi-step screening platform, the authors report the best performing nanoparticle formulations for liver-targeted plasmid DNA expression in vivo.

## Introduction

Development of delivery systems and methods remain the most important challenge in realizing the tremendous potential of delivering nucleic acids for gene therapy. RNA- and DNA-based biologics have expansive capacities to modulate cellular activities for treating inherited and acquired diseases^1^. Among the non-viral gene delivery vectors, the clinical success of LNPs has gained recent widespread attention^2,3^. This is highlighted by the US Food and Drug Administration (FDA)-approved short interfering RNA therapy for hereditary amyloidosis (ONPATTRO^®^, patisiran) and the two mRNA COVID-19 vaccines approved or authorized for emergency use by millions of healthy people during the pandemic^4–6^. Most lipid-based nucleic acid delivery platforms that are undergoing clinical studies or on the market consist of four or five components: an ionizable lipid, cholesterol, a PEGylated lipid, a helper phospholipid (e.g., 1,2-distearoyl-*sn*-glycero-3-phosphocholine (DSPC)), and a selective organ targeting lipid^7–10^. Recent studies have reported that not only the choice of lipid components, but also the relative proportions of the lipid ingredients in the formulation, greatly influence in vivo transfection efficiency and tissue-specific delivery^7,11–14^. Despite the validated ability of these formulations to encapsulate mRNA or siRNA and mediate cellular uptake and endosomal escape, there is a lack of in-depth analysis on the effect of helper lipid charge and the relative ratios of the LNP components on the transfection efficiency for plasmid DNA (pDNA) delivery, which can provide prolonged transgene expression compared to mRNA^15–18^. In addition, the large number of candidate formulations for screening LNP systems for effective in vivo delivery makes it difficult to rationally determine the optimal formulation for specific tissue or disease targets.

**Fig. 1:**
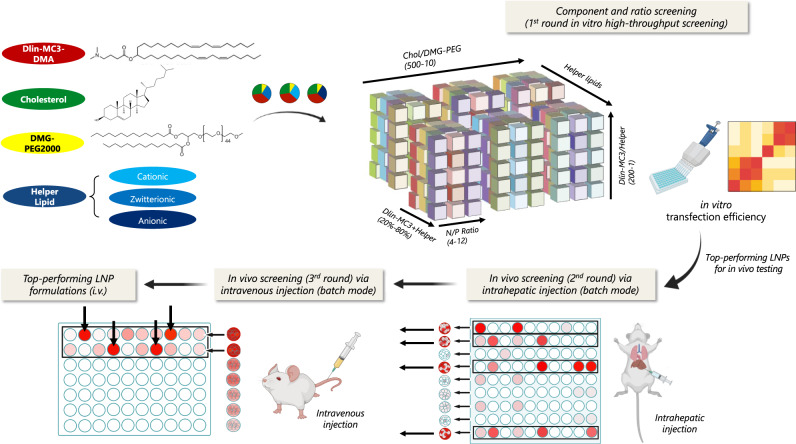
Schematic illustration of multi-step composition screening of lipid nanoparticles (LNPs) for liver-targeted pDNA delivery. In vitro transfection efficiency was assessed for 1080 LNP formulations with different helper lipids and component ratios. The top-performing formulations for each helper lipid series were then tested in clusters for cytotoxicity and in vivo local transfection efficiency via intrahepatic injection. Clusters that induced minimal cytotoxicity and high transfection were screened via i.v. injection, and LNP formulations within the clusters that demonstrated optimal liver transfection were further evaluated individually.

Herein, we report a multi-step screening platform to systematically test and analyze the liver-targeted transfection efficiency of 1080 LNP formulations with different helper lipids and component ratios in vitro and in vivo (Fig. 1). In general, a cohort of formulations that delivered the highest levels of in vitro transfection efficiency were identified first via high-throughput screening. Inspired by the pooled diagnostic testing methods widely used during the COVID-19 pandemic, a cluster-mode screening approach was used in the initial in vivo screening step in groups of eight. These clusters were initially screened via intrahepatic injection to assess local toxicity and transgene expression levels. Clusters with minimal cytotoxicity and the highest transfection efficiencies were then selected for intravenous (i.v.) injection testing; and formulations within the clusters that demonstrated optimal liver transfection were further individually evaluated for i.v. injections. Using this multi-step composition screening platform, we aimed to identify the most efficient pDNA LNP formulations from the designed library for liver-targeted transfection via i.v. administration. We also compared the transgene expression level and duration of the optimized pDNA LNPs with the widely used pDNA/PEI nanoparticles and mRNA LNPs^19^. To further understand the rate-limiting steps of the in vivo gene delivery process for pDNA LNPs, we examined the in vivo biodistribution profile, cellular uptake level, and lysosome escape capability for the top-performing formulations and compared them with formulations that were less effective but shared similar characteristics.

Another challenge that must be overcome to fully realize the potential of LNP-mediated pDNA delivery is immune-mediated silencing of the transgene^20–25^. Several approaches have been explored to extend expression duration and reduce immune response, including sequence modification to reduce CpG island density and optimization of delivery carriers^16,20^. To further improve the delivery efficiency of the top formulations, we describe a new siRNA co-delivery strategy that targets key transcription factors regulating inflammatory response pathways to reduce inflammation-induced gene silencing. Using an optimized LNP formulation, we examined the effect of co-delivering pDNA and siRNAs against signal transducer and activator of transcription (STAT)^26^ and nuclear factor kappa-light-chain-enhancer of activated B cells (NF-κB)^27–29^ on the level and duration of transgene expression following i.v. administration.

## Results

### Design of an LNP library for the screening of LNP-mediated pDNA delivery

DLin-MC3-DMA was selected as the ionizable lipid, and DMG-PEG2000 was used as the PEGylated lipid. Six helper phospholipids previously used in experimental or FDA-approved LNP formulations were chosen to represent a range of charges for testing: the cationic 1,2-dioleoyl-3-trimethylammonium-propane (DOTAP) and dimethyldioctadecyl ammonium (DDAB); the zwitterionic 1,2-dioleoyl-*sn*-glycero-3-phosphoethanolamine (DOPE) and DSPC; and the anionic 1,2-dimyristoyl-sn-glycero-3-phosphate (14PA) and 1-stearoyl-2-oleoyl-sn-glycero-3-phospho-(1’-rac-glycerol) (18PG)^6,7,10^. Using DLin-MC3-DMA, cholesterol, DMG-PEG2000, and one of the six helper lipids, we designed an initial library of 1080 LNP formulations by varying the following parameters: (1) ratio of DLin-MC3-DMA to helper lipid ranging from 1 to 200; (2) ratio of cholesterol to DMG-PEG2000 ranging from 10 to 500; (3) combined percentage of DLin-MC3-DMA and helper lipid ranging from 20% to 80%; and (4) N/P ratio ranging from 4 to 12. This parameter design provided us with a sufficiently diverse library of LNP formulations, with which we programmatically tested LNP-mediated pDNA delivery.

**Fig. 2:**
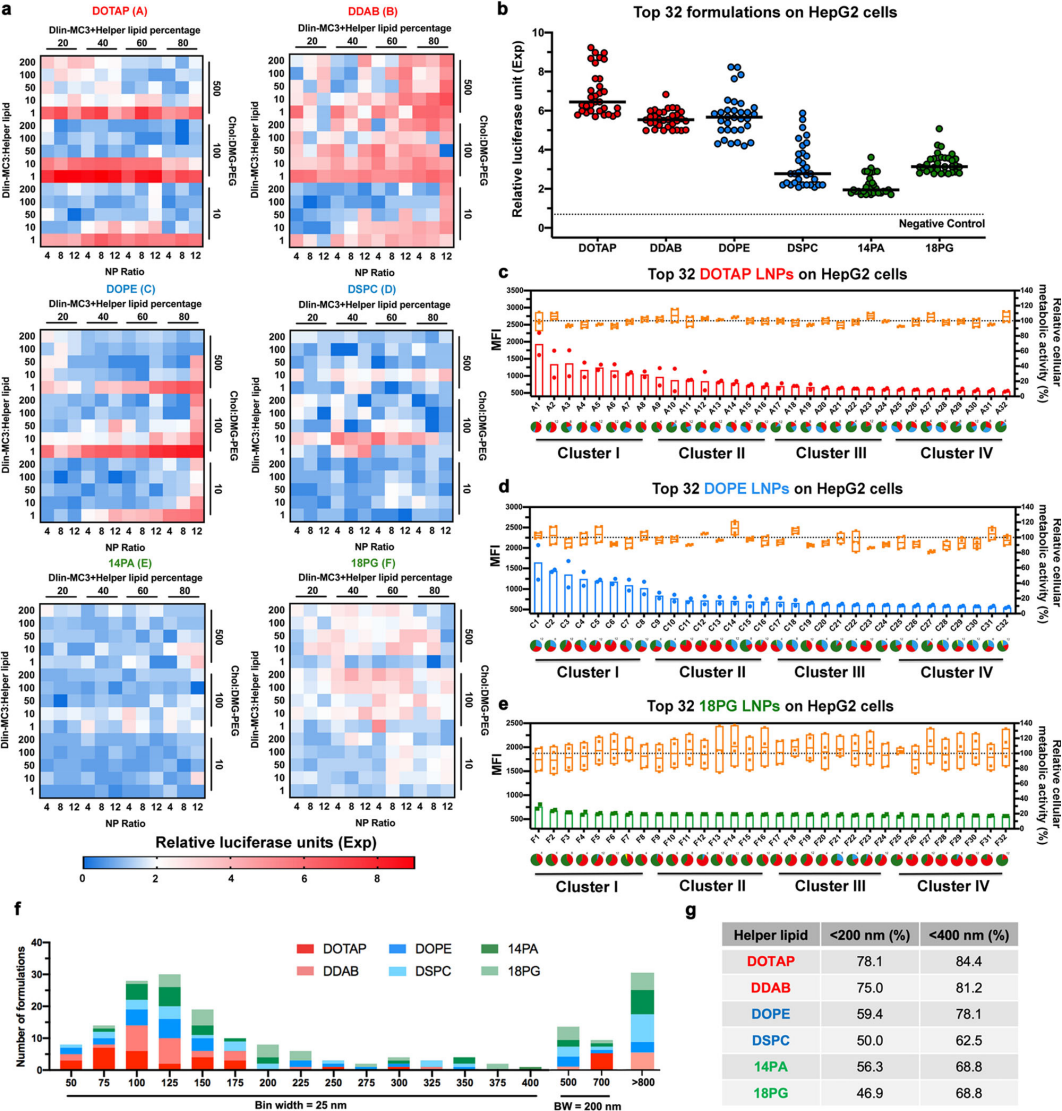
In vitro LNP-mediated pDNA delivery. **a** Transfection efficiency of LNPs in HepG2 cells via high-throughput screening platform after 72 h incubation (*n* = 2). The level of transgene expression for each formulation is shown using luciferase as a reporter. **b** The top 32 formulations from each helper lipid series were selected based on transfection efficiency in HepG2. FACS was used to further evaluate the transfection efficiency of **c** DOTAP, **d** DOPE and **e** 18PG series of LNPs using GFP as a reporter gene at a pDNA dose of 1 μg/mL; gene expression was analyzed at 72 h after transfection (*n* = 2). Cellular metabolic activity was measured by alamarBlue assay (*n* = 4). Formulations were regrouped into four clusters, each containing eight formulations, based on their transgene expression level. Data are presented as mean±S.D. The percentage of each component in the formulations is indicated by pie charts: Ionizable lipid (red), cholesterol (green), helper lipid (blue), DMG-PEG2000 (yellow). See Supplementary Tables 1–6 for molar percentage used in the 32 formulations for each helper lipid. Bars refer to the MFI (Median fluorescence intensity) value on the left, the boxes refer to the metabolic activity on the right. **f** Histogram of the Z-average diameters of top 32 LNP formulations made from each helper lipid. **g** Percentage of LNP formulations with size less than 200 nm and less than 400 nm for each helper lipid. Source data are provided as a Source Data file.

To select LNP formulations with strong liver-specific transgene expression, we first evaluated the pDNA delivery efficiency of the whole library using firefly luciferase pDNA and measured luciferase protein expression in HepG2 cells (a human liver cancer cell line) (Fig. 2a). When fixing the helper lipid, adjusting the above-mentioned four parameters in the LNP formulations significantly varied the gene expression levels. Of the 1080 LNP formulations, the 32 top-performing LNPs for each helper lipid group are shown in Fig. 2b. Next, we examined the cytotoxicity and re-evaluated the transfection efficiency of the top 32 formulations via flow cytometry analysis. Results shown in Fig. 2c–e and Supplementary Fig. 1 confirmed the high in vitro transfection efficiency and good biocompatibility of these LNPs. Compared with a commonly used liposome transfection agent, lipofectamine 3000 (Supplementary Fig. 1d, metabolic activity was 53.13 ± 9.59%), only minimal cytotoxicity exerted on treated cells was observed for the selected top 32 LNP formulations (overall metabolic activity was 96.35 ± 7.18%). We further measured the average sizes and size distributions of top-performing LNPs using dynamic light scattering (DLS) (Fig. 2f). Results indicated most of those LNPs had a size smaller than 400 nm (~73.9%), and for cationic helper lipids, the percentage of smaller LNPs (<400 nm) were higher (~80%) compared to others (e.g., ~68% for anionic helper lipids) (Fig. 2g). Through detailed analysis, some correlations between the average size and formulation parameters were observed: (a) As the N/P ratio increased from 4 to 12, the average size became more uniform (*i.e*. with lower standard deviation) (Supplementary Fig. 2a); (b) As the cholesterol content (molar percentage) increased from ~20% to ~80%, the average LNP size increased, but there was no significant change in polydispersity index (PDI) (Supplementary Fig. 2b); and (c) As the DMG-PEG2000 content (molar percentage) increased from ~0.03% to ~5.5%, the LNP size became more uniform (Supplementary Fig. 2c).

### LNP-mediated intrahepatic pDNA delivery via cluster-mode in vivo screening

**Fig. 3:**
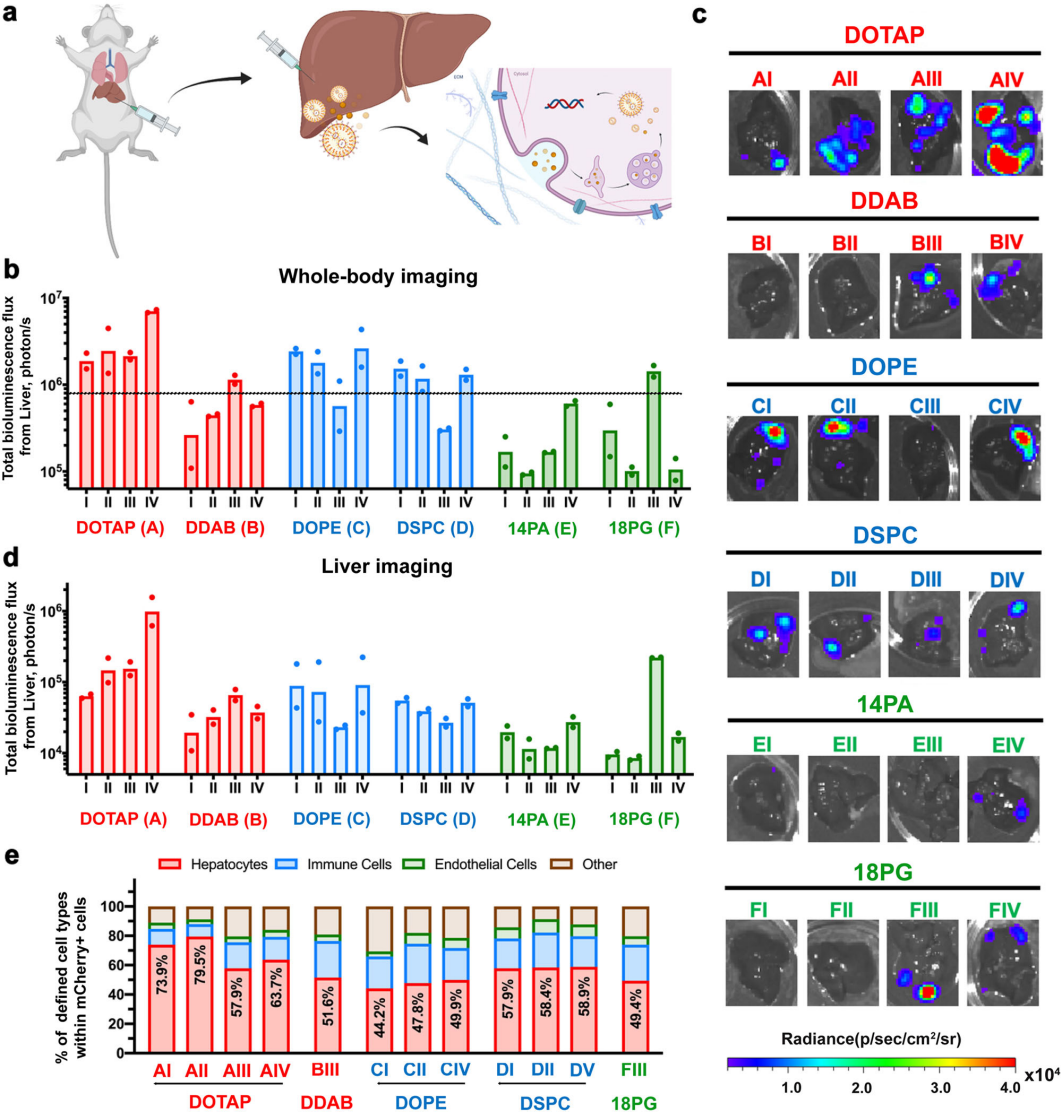
LNP-mediated local intrahepatic pDNA delivery in cluster-mode screening. **a** Scheme for intrahepatic delivery. **b** Whole-body bioluminescence flux of female BALB/c mice (6–8 weeks) at 12 h following a single intrahepatic injection of different LNP formulations containing 3 μg of pDNA (50% Luc+ 50% mCherry) per mouse (*n* = 2, 48 for the whole experiment). Ex vivo **c** imaging and **d** quantitative flux of luminescence in the liver at 12 h post-administration. **e** FACS was used to quantify the percentage of specific cell types in the liver expressing mCherry for the top 12 clusters. Data are presented as mean±S.D. (*n* = 2 mice per group). Source data are provided as a Source Data file.

The in vivo transfection efficiency of LNPs likely differs from that of traditional in vitro assay screens due to the difference between in vivo and in vitro settings and delivery barriers. To evaluate their local in vivo tissue transfection efficiency, the LNPs that showed the highest range of in vitro transfection efficiency were tested by intrahepatic injection (Fig. 3a). As previously mentioned, a cluster-mode screening method was used in this in vivo screening process, greatly reducing the number of animals, time, and cost required.

We first grouped the top-performing LNP formulations into four clusters for each helper lipid (in total 24 clusters and eight formulations per cluster) based on in vitro transfection efficiency (Fig. 2c–e and Supplementary Fig. 1), as one of the possible factors that can be used for clustering the formulations. Alternatively, it is possible to choose a variety of different clustering methods in such a cluster-mode screening approach including structure of the components such as size, surface charge, or levels of certain lipid components, in vitro transfection efficiency, and even randomization methods^14,30–32^. The effects of each cluster were examined by delivering a combination of two plasmids, luciferase (Luc) (50%) and mCherry (50%) pDNA, at a total dose of 3 μg pDNA per mouse via intrahepatic injection. Unexpectedly, clusters with high transfection efficiency in vitro were not necessarily the top-performing clusters in vivo; and this finding applied to both clusters that included the same helper lipid and all 24 clusters (Fig. 3b–d). For example, cluster AIV, which contained the eight DOTAP formulations with the lowest in vitro transfection efficiency in the DOTAP group, produced an average bioluminescence signal (Luc expression) 17.2 times higher than that of cluster AI (composed of the eight top-performing DOTAP formulations in vitro) (Fig. 3d). Moreover, clusters that showed a moderate efficiency in vitro could have potent transfection efficiency in vivo. For instance, cluster FIII had a surprisingly high transfection efficiency in contrast to its in vitro performance. In addition, the charge of the helper lipids significantly influenced the transfection efficiency; cationic lipids like DOTAP had a more potent effect than others, especially compared to the least effective anionic lipid, 14PA. However, although DOTAP and DDAB are both cationic lipids, the local transfection of DDAB clusters (such as BI, BII, and BIV) in the liver was low. Similarly, most clusters composed of anionic helper lipids had limited local transfection in the liver, but there was a unique cluster (FIII) in the 18PG group that achieved relatively high transfection efficiency. Likewise, although DOPE and DSPC clusters had high transfection efficiency overall, clusters CIII and DIII showed lower efficiency. No significant luciferase expression was detected in othertissues of the sele based on the whole-body imaging analysis.

To gain an initial estimate of the cell types transfected by the 12 top-performing clusters (shown in Fig. 3b above the dashed line), we quantified mCherry expression levels in various cell types within the liver using flow cytometry (Fig. 3e). After a single intrahepatic injection, in all top 12 clusters, around 50% of the transfected cells in the liver were hepatocytes followed by the second majority of immune cells. Particularly, for cluster AI and AII, the transfection efficiency in hepatocytes reached as high as 73.9% and 79.5%, respectively. This result showed that these clusters contained LNP formulations that could have potent liver-specific transfection. Based on the IVIS results (Fig. 3b and d), we successfully identified the top 12 clusters of LNPs (96 formulations in total) as candidate clusters for further evaluation of their stability within blood circulation, and tissue-specific transfection efficiency following systemic delivery.

### LNP-mediated, liver-specific pDNA delivery via cluster-mode testing

**Fig. 4:**
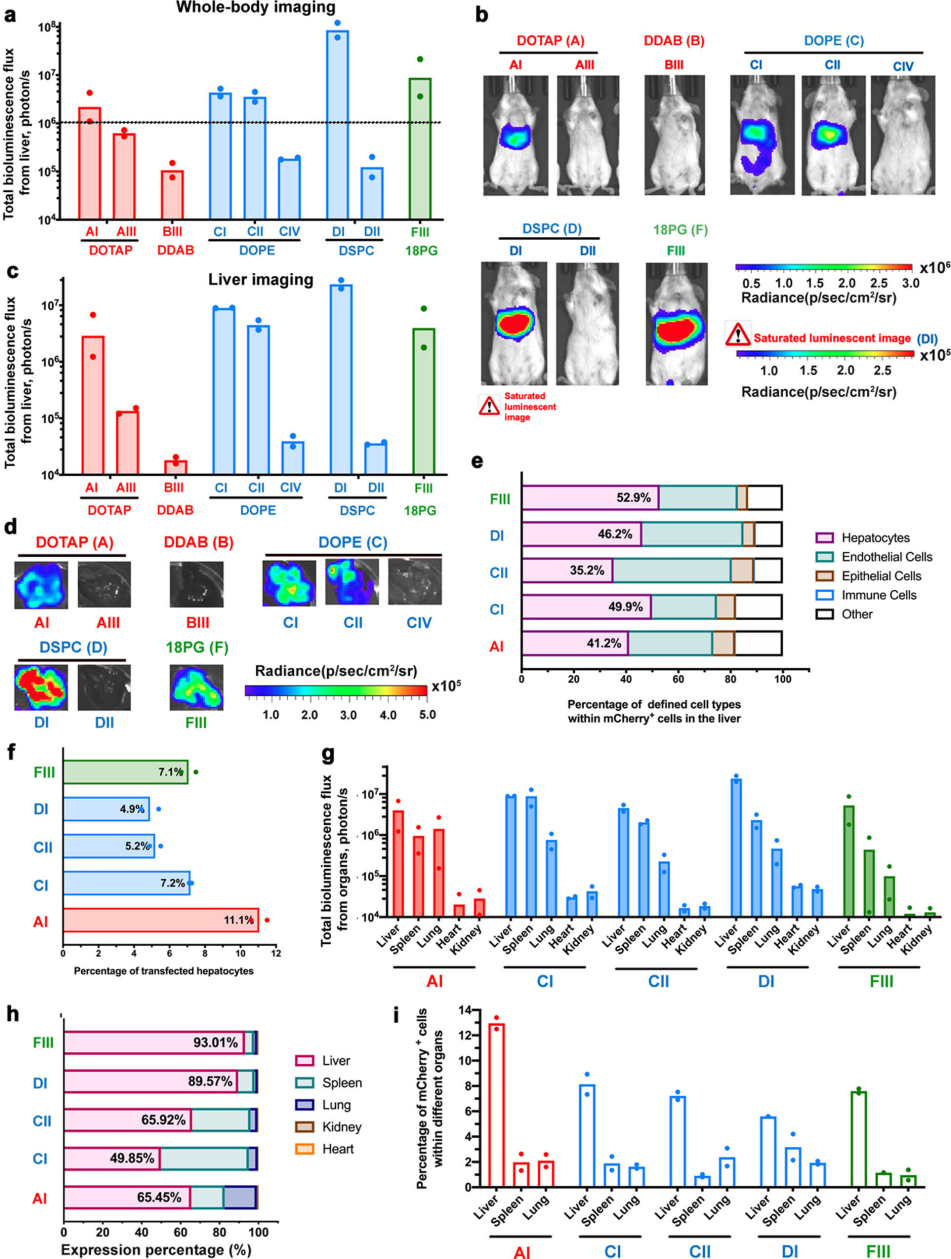
In vivo transfection efficiency of LNPs administered via *i.v*. injection in cluster-mode screening. Whole-body bioluminescence **a** quantitative measurement and **b** imaging of female BALB/c mice (6–8 weeks) at 12 h after a single i.v. administration of different LNPs formulation containing 100 μg of pDNA (50% Luc+50% mCherry) per mouse (*n* = 2, 24 mice in total). **c**, **d** Ex vivo imaging and quantitative luminescence measurement of the liver of BALB/c mice at 12 h post-administration. (*n* = 2) **e** FACS was used to quantify the percentage of specific cell types within mCherry+ cells in the liver. (*n* = 2) **f** FACS was used to quantify the percentage of mCherry+ cells within hepatocytes (FSC^hi^ SSC^hi^ cells in CD45^-^CD31^-^CD11b^-^CD326^-^ cells). (*n* = 2) **g**, **h** Quantitative measurement of luminescence and relative luciferase expression in each organ. (*n* = 2) **i** FACS was used to quantify the percentage of mCherry+ cells within the major organs. (*n* = 2) Data above are presented as mean±S.D. Source data are provided as a Source Data file.

The 12 clusters that demonstrated the highest transgene expression levels in the liver were then examined for performance via the i.v route. The same payload, luciferase (Luc) (50%) and mCherry (50%) pDNA, were encapsulated in these LNPs, which were administered intravenously at a total pDNA dose of 100 μg per mouse. Three clusters (AII, AIV, and DIV) among the 12 tested showed significant toxicity after i.v administration and were excluded from further evaluation (Supplementary Fig. 3). Five clusters (AI, CI, CII, DI and FIII) were the most efficient clusters for liver-specific transgene expression (Fig. 4a–d). Compared with other clusters, these five clusters gave an average of 2 to 3 orders of magnitude higher luciferase expression in the liver at 12 h after i.v. administration measured by IVIS imaging. For example, cluster DI was 660 times higher than that of cluster DII. For these top five clusters of LNPs, we further quantified delivery to specific cell types within the liver using flow cytometry to detect mCherry expression, which revealed that about 40% of transfected cells in the liver were hepatocytes and about 7% of the total hepatocytes in the liver were successfully transfected (Fig. 4e, f).

Next, we evaluated the transgene expression level of the top five clusters in othertissues of the sele including the spleen, lung, kidney, and heart (Fig. 4g). Based on the relative Luc expression in each organ, two clusters, DI and FIII, yielded high liver-specific transfection efficiency; 89.6% of bioluminescence among thetissues of the sele was from the liver for DI, and 93.0% for FIII (Fig. 4h). In addition, higher levels of transgene expression in the spleen were observed for clusters CI (45.2%) and CII (30.0%). The percentage of transfected cells in three majortissues of the sele (liver, spleen, and lung) were further evaluated through flow cytometry; for all five clusters, roughly 10% of cells in the liver were transfected based on mCherry expression (Fig. 4i). Based on these data, clusters DI and FIII were selected for further characterization.

### Formulations for liver-specific pDNA delivery

**Fig. 5:**
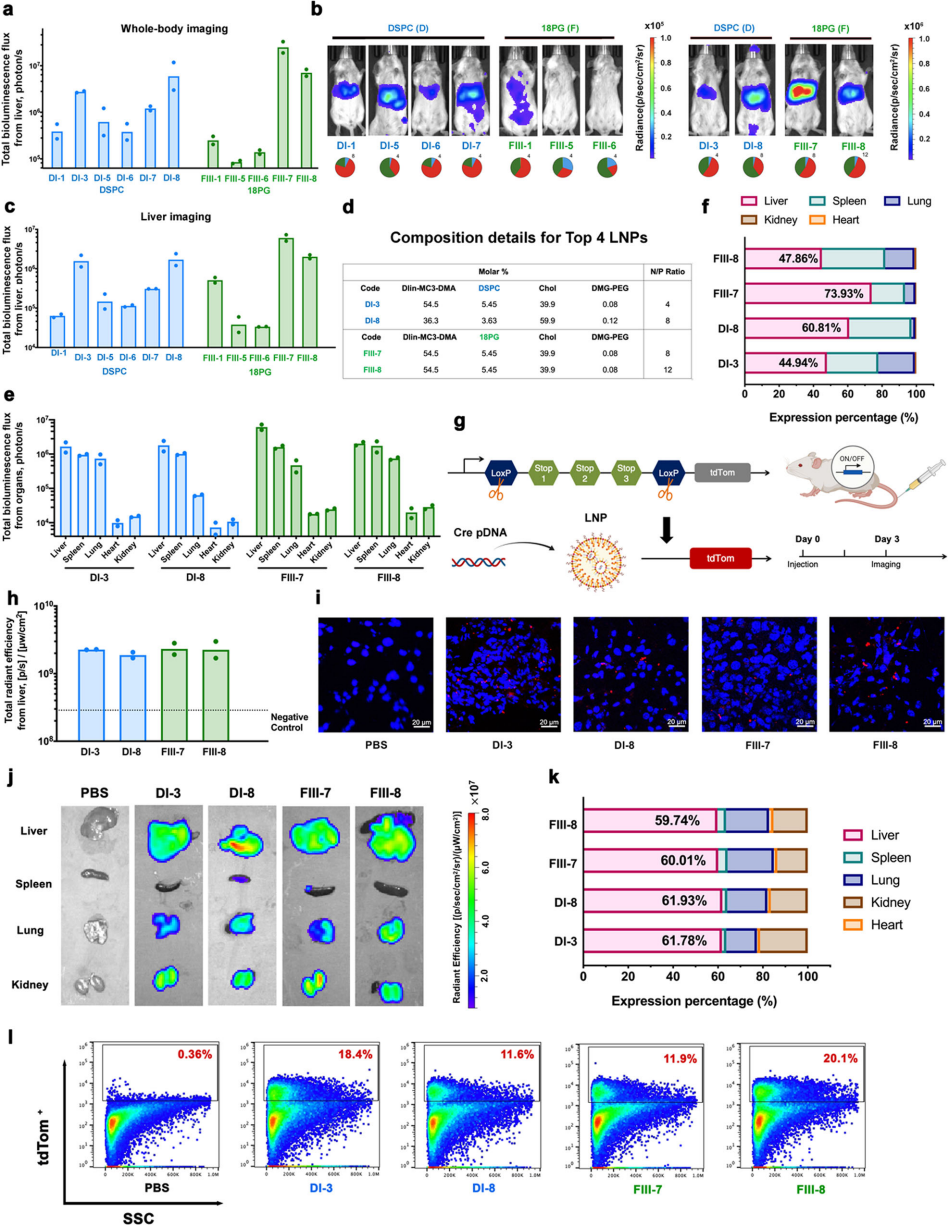
Liver-targeted transfection efficiency and in vivo gene editing by LNPs administered via *i.v*. injection. **a**, **b** Whole-body bioluminescence imaging and quantitative measurement of female BALB/c mice (6–8 weeks) at 12 h after a single i.v. injection of different LNP formulations containing 50 μg of pDNA (50% Luc+50% mCherry) per mouse (*n* = 2, 32 mice in total). **c** Ex vivo imaging and quantitative luminescence measurement of the liver of BALB/c mice at 12 h post-administration with single dosage. (*n* = 2) **d** Composition details for the top four LNP formulations. **e**, **f** Quantitative measurement of luminescence and relative luciferase expression level in each organ. (*n* = 2) **g** Schematic illustrating that the delivery of Cre pDNA activates tdTom expression in tdTom transgenic mice via Cre-mediated genetic deletion of the stop cassette. **h** Ex vivo quantitative measurement of tdTom fluorescence in the liver of female Ai9 mice (6–8 weeks) at 3 days post-administration with a single dosage of LNPs containing 25 μg of pDNA (Cre) per mouse (*i.v*., *n* = 2, 10 mice in total). **i** TdTom expression visualized by confocal imaging of tissue sections. **j** Ex vivo imaging of tdTom fluorescence in the different organs. (*n* = 2) **k** Relative tdTom expression in each organ. (*n* = 2) **l** FACS was used to quantify the percentage of tdTom+ cells in the liver. All data are presented as mean±S.D. Source data are provided as a Source Data file.

The transfection efficiencies of the 16 individual LNP formulations within the DI and FIII clusters were further examined following i.v. injection at a total pDNA dose of 50 μg per mouse using the same Luc/mCherry combination (50/50) payload. In the 8-formulation cluster testing, we tested a total dose of 100 µg pDNA per mouse for i.v. injection, which corresponded to a 12.5 μg pDNA dose for each individual LNP formulation. When we evaluated the individual formulations in the validation experiment, we selected a dosage of 25 μg Luc pDNA per mouse, to confirm the effect at a double dose for an individual formulation, but four-times lower than the total dose for the cluster. Five of the 16 formulations (DI-2, DI-4, FIII-2, FIII-3 and FIII-4) showed high toxicity following i.v. administration and were excluded from further evaluation (Supplementary Fig. 4). Based on IVIS results shown in Fig. 5a–c, four individual formulations (DI-3, DI-8, FIII-7, and FIII-8, Fig. 5d) showed the highest levels of Luc expression in the liver. The best-performing formulation, FIII-7, demonstrated a 300-fold higher Luc expression than FIII-5, which is another formulation within the same cluster. The transgene expression levels in other majortissues of the sele mediated by the top four formulations were also evaluated (Fig. 5e). Of the total bioluminescence among various organs, 73.9% occurred in the liver for FIII-7 and 60.8% for DI-8, with both formulations showing a high-level of liver-specific transgene expression (Fig. 5f). These top four formulations (DI-3, DI-8, FIII-7, and FIII-8) were therefore advanced to further testing using an orthogonal assay to measure liver-specific transgene expression.

### Validation of top formulations for liver-specific pDNA delivery

To further validate the efficiencies of the top four LNP formulations in mediating liver-specific gene delivery, we utilized genetically engineered tdTom reporter mice (Ai9 mice) containing a LoxP-flanked stop cassette that prevents expression of the tdTom protein. This mouse model allows detection of the gene-edited cells as a result of Cre expression (Fig. 5g). When Cre recombinase is introduced into the reporter mouse cells, it recombines the DNA at the LoxP sites to excise the stop cassette, which permits the expression of fluorescent tdTom. We used the four formulations (DI-3, DI-8, FIII-7, and FIII-8) to deliver Cre recombinase pDNA following i.v. injection (25 μg pDNA/mouse). Three days post injection, a high tdTom signal was detected in the liver (Fig. 5h); and tdTom-positive cells were easily observed using confocal imaging of tissue sections (Fig. 5i). Fluorescent signal was also observed in other organs (Fig. 5j, Supplementary Fig. 5), but about 60% of the total tdTom expression among imaged organs was from the liver for all four LNPs (Fig. 5k). We used flow cytometry to further quantify the percentage of gene-edited cells in the liver and found that about 20% of the cells were successfully edited by treatment with FIII-8 (Fig. 5l, Supplementary Fig. 6). Based on high transfection efficiency and high biocompatibility, the top four LNPs may be applicable to liver-targeting gene therapy via systemic delivery.

Additionally, a difference in terms of transgene level in different organs was observed between two reporter systems (Luc pDNA and Cre pDNA). This difference was likely caused not only by different imaging time points but also the difference between transgene processes. For the Cre-Ai9 mouse model used in Fig. 5g–l, Cre recombinase needs to be expressed first, enters the nucleus and then catalyzes Lox-specific DNA recombination, which induces robust tdTom expression. The whole process is different from the luciferase expression model where the Luc pDNA was transcribed and expressed after successful transfection.

### Biodistribution, cellular uptake and endosomal escape level of top-performing formulations

Despite having successfully identified the top four LNP formulations using the multi-step screening platform, the mechanism still remained unclear. Therefore, we proposed the following hypotheses: enhanced liver-targeting transfection is the result of (1) the tissue-specific biodistribution of LNPs, (2) the differential cellular uptake profiles of LNPs following distribution into the local tissue, and (3) the differential endosomal escape or DNA release abilities of LNPs, even between formulations with similar biodistribution and cellular uptake levels.

**Fig. 6:**
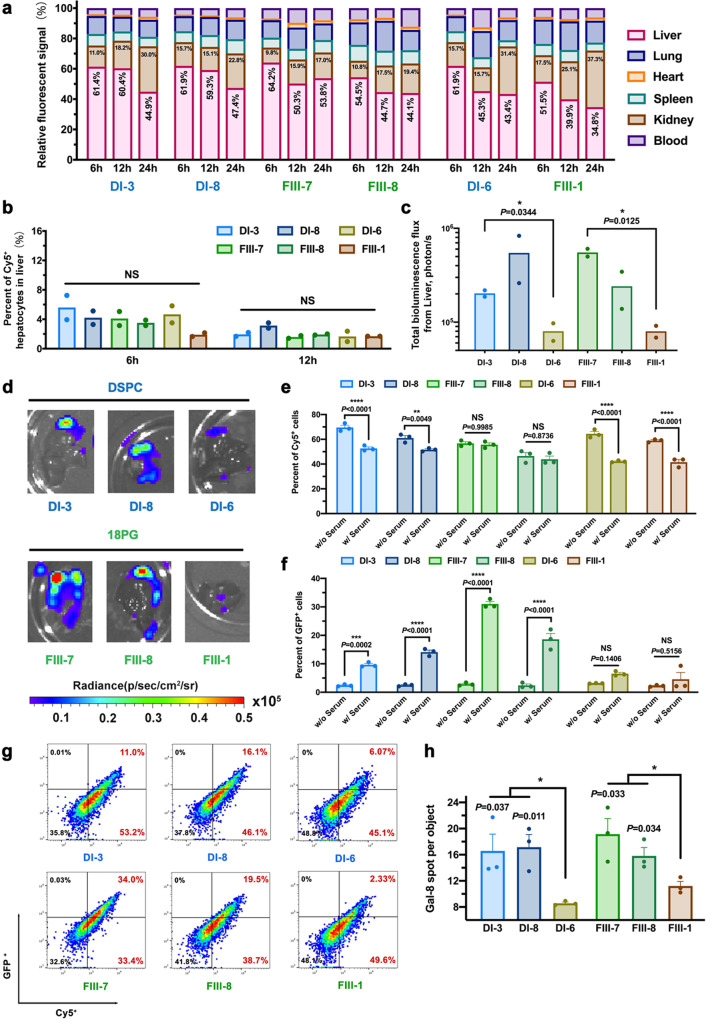
Biodistribution, cellular uptake and endosomal escape levels of top-performing LNP formulations. **a** Biodistribution at 6, 12 and 24 h post-injection of LNPs (30 μg pDNA (85% Luc+15% Cy5-labeled p1216) per mouse. i.v. *n* = 2, 36 female BALB/c mice (6–8 weeks) in total). **b** FACS was used to quantify the percentage of Cy5+ hepatocytes in the liver at 6 and 12 h post-injection. (*n* = 2) **c**, **d** Ex vivo quantitative measurement and luminescence imaging of the liver of BALB/c mice at 24 h post-administration (25 μg Luc pDNA per mouse, intrahepatic injection, *n* = 2). **e**–**g** In vitro transfection and cellular uptake of selected formulations on primary hepatocytes. FACS was used to quantify the percentages of **e** Cy5+ cells and **f** GFP+ cells within primary hepatocytes isolated from the liver (1 μg/mL pDNA (75% GFP + 25% Cy5-labeled p1216)). (*n* = 3) Data are presented as mean values + /− SEM. **g** Representative FACS data for LNPs pre-incubated with mouse serum for 0.5 h at an LNP/serum volume ratio of 2:1 before dosing. **h** Quantitative Cellomics high-content analysis for endosomal escape mediated by LNPs in vitro. Average number of Gal8 spots per cell (B16-Gal8-GFP) at 12 h post-treatment as an indication of endosomal escape level (1 μg/mL pDNA) (*n* = 3). Data are presented as mean values + /− SEM. Data were analyzed using one-way ANOVA and Tukey’s multiple comparisons test (two-sided) for Fig. 6b, c, e, f, h. Statistical *P*-values: No significance: NS; ^*^P < 0.05, ^**^P < 0.01, ^***^P < 0.001, ^****^P < 0.0001. Source data are provided as a Source Data file.

To test our hypotheses, we first examined the serum stability and biodistribution of the four top-performing LNPs at 6, 12, and 24 h after i.v. injection using Cy5-labeled pDNA. For comparison, we included two LNP formulations (DI-6 and FIII-1) that were less effective but possessed similar characteristics (size and zeta potential) to the top-performing ones (Supplementary Fig. 7). As Supplementary Fig. 8 showed, there was no significant difference between top-performing LNPs and low-performing ones observed in terms of size change in serum containing PBS. IVIS imaging showed that all six selected formulations, independent of transfection performance, had similar biodistribution profiles at all time points (Fig. 6a, Supplementary Fig. 9). In all cases, ~60% of LNPs were distributed to the liver at 6 h after i.v. administration. In addition, similar uptake levels by hepatocytes were seen for all six formulations, with flow cytometry assessment indicating that the high transfection by the top formulations was not caused by differences in biodistribution nor cellular uptake level (Fig. 6b). To verify that the transfection efficiency of LNPs was not related to biodistribution, we checked the transfection efficiency of six LNPs by administering the same dose of the six LNPs via intrahepatic injection. The results in Fig. 6c and d showed that although the same dosage was delivered to the liver, the local transfection efficiency was significantly different. Compared with DI-6 and FIII-1, the top four formulations indeed provided a higher transfection efficiency. Thus, regardless of the delivery route, transfection efficiency was not strictly related to biodistribution.

To verify that endosomal escape and/or DNA release improved the transfection efficiency of LNP formulations, primary mouse hepatocytes were isolated and transfected with the six LNPs (DI-3, DI-8, FIII-7, FIII-8, DI-6, and FIII-1) using 75% GFP + 25% Cy5-labeled pDNA. To simulate the situation in vivo, LNPs were incubated with fresh mouse serum at a 2:1 volume ratio (LNP/serum) at 37 °C for 30 min before dosing to cells. With or without pre-incubation with mouse serum, all six LNPs exhibited similar uptake levels, which is consistent with the in vivo cellular uptake level observed (Fig. 6e). While pre-incubation with mouse serum did not significantly impact cellular uptake level, the transfection efficiency of the best four LNPs was greatly improved; the percentage of GFP positive cells increased by 12 times after serum pre-incubation for FIII-7 (Fig. 6f and g), whereas no significant difference was observed in the transfection efficiencies of DI-6 and FIII-1 compared to the drastic increases for the top four formulations. Furthermore, the endosomal escape capability of selected formulations was examined by quantitative Cellomics high-content analysis on an established B16-Gal8-GFP cell line^33,34^. The results showed in Fig. 6h indicated that the top four formulations (DI-3, DI-8, FIII-7, and FIII-8) have a relatively higher endosomal escape capacity.

The above results showed that LNPs with similar size and zeta potential yielded a similar biodistribution and cellular uptake profile after i.v. injection; the varied endosomal escape capability of different LNP formulations was likely the determining factor for difference in the transfection efficiency. Moreover, the significant difference observed between groups incubated with or without serum suggested that the discrepancies between in vivo and in vitro experiments may be closely related to serum-mediated opsonization of the LNPs.

### Extending transgene expression duration of LNPs by co-delivery with anti-inflammatory siRNAs

**Fig. 7:**
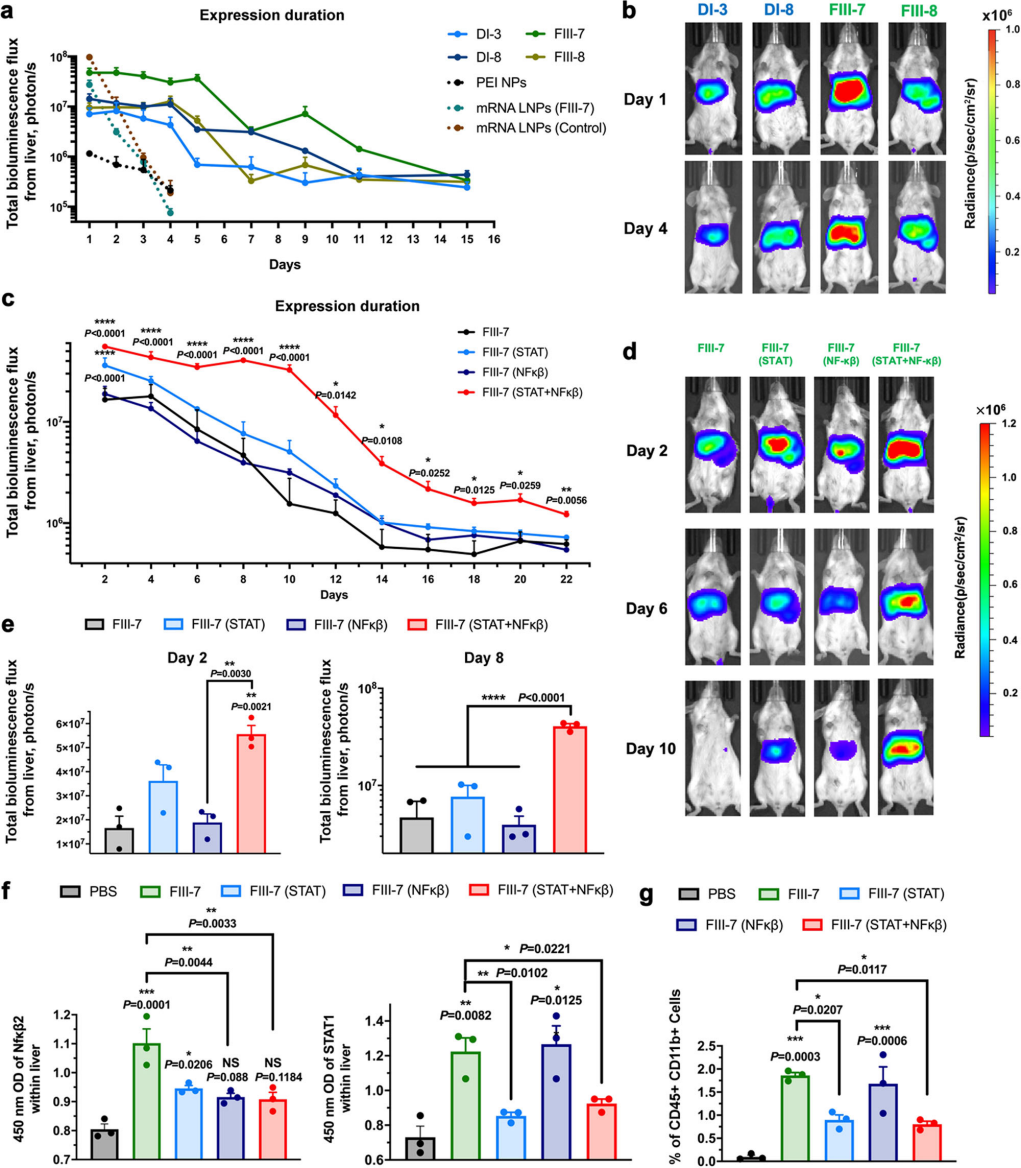
More durable expression of pDNA LNPs and extended transgene expression duration by co-delivery of anti-inflammatory siRNA. **a**, **b** Whole-body bioluminescence imaging of female BALB/c mice (6–8 weeks) at different time points after i.v. administration of a single dosage of LNPs containing 25 μg of Luc pDNA per mouse or 5 μg of Luc mRNA per mouse for mRNA LNPs (*n* = 3, 21 mice in total). A mRNA LNP with the same composition as the ONPATTRO® was used as a control. **c**–**e** Whole-body bioluminescence imaging of female BALB/c mice (6–8 weeks) at different time points post-administration (25 μg Luc pDNA per mouse, 2.5 μg siRNA for each transcription factor per mouse (*n* = 3, 12 mice in total). **f** The levels of transcription factors of treated mice were determined by ELISA at 7 days post-administration with single dosage. (female BALB/c mice (6–8 weeks), *n* = 3, 15 mice in total) **g** FACScan was used to determine the infiltrating inflammatory monocytes (CD45^+^CD11b^+^ cells) in the liver after treatments. (*n* = 3) Data above are presented as mean±S.E.M. Data were analyzed using one-way ANOVA and Tukey’s multiple comparisons test (two-sided) for Fig. 7c, e, f and g. Statistical *P*-values: No significance: NS; ^*^P < 0.05, ^**^P < 0.01, ^***^P < 0.001, ^****^P < 0.0001. Without specific indications, the label above each group indicates the statistical comparison with the PBS control group. Source data are provided as a Source Data file.

The duration of expression within the liver in BALB/c mice was monitored following the i.v. injection of the top four formulations (DI-3, DI-8, FIII-7, and FIII-8) (Fig. 7a and b). The initial expression levels of the four formulations were consistent with data shown above, and the expression was maintained at a similar level for about 4 days, before declining over 3 to 7 days after that. Two control groups loaded with Luc mRNA using FIII-7 LNP formulation and an LNP formulation with the same composition as ONPATTRO^®^ were also tested (5 μg mRNA per mice, i.v.), and showed a significantly reduced expression duration relative to LNPs containing pDNA. Although the initial expression within the liver by mRNA LNPs was comparable on day 1 to that of pDNA LNPs (25 µg pDNA/mouse), the expression level dropped by 10-fold on day 2 and decreased by more than 300-fold on day 4. A polycationic carrier Polyplu*s* in vivo-jetPEI was used to generate pDNA/PEI nanoparticles (PEI NPs), and was also tested for comparison. The majority of Luc expression level mediated by PEI NPs were found in the lung, and transgene expression in the liver was much lower than FIII-7 pDNA LNPs (~2.4%) on day 1^19^. Thus, pDNA LNPs provided substantially longer transgene expression than either of the tested comparators.

To further extend the expression of pDNA, several methods are being explored to reduce undesirable innate immune activation and host toxicity such as sequence modification (CpG-depleted pDNA) and delivery vector optimization^16,20^. Here, we test the effect of co-delivering anti-inflammatory siRNAs against signal transducer and activator of transcription-1 (STAT1) and nuclear factor kappa-light-chain-enhancer of activated B cells −2(NF-κB2) to prolong the duration of expression mediated by pDNA co-packaged in the same LNPs^26,27,29,35,36^. For these experiments, we used 2.5 μg siRNA/mouse for each transcription factor. As Fig. 7c–e showed, the luciferase expression mediated by FIII-7 LNPs persisted for ten days when siRNA against STAT1 and NF-κB2 were co-delivered, in contrast to five days when pDNA was delivered alone with the same LNPs. In addition, the initial expression levels of FIII-7 (STAT1 + NF-κB2) LNPs and FIII-7 (STAT1) LNPs within the liver were 3.4- and 2.2-fold higher on day 2, respectively, than that of FIII-7 LNPs alone (Fig. 7e). At 7 days after administration, the STAT1 and NF-κB2 level within the liver was examined by ELISA. As Fig. 7f shows, the elevated levels of both inflammatory transcription factors were observed after i.v. injection of FIII-7 LNPs; and codelivery with anti-inflammatory siRNAs significantly reduced the transcription factor levels. In the FIII-7 (STAT1 + NF-κB2) LNP group, the levels of NF-κB2 and STAT1 were reduced by ~17.6% and ~24.5%, respectively, compared with injections with FIII-7 LNPs. In addition, lower levels of infiltrating inflammatory monocytes (CD45^+^ CD11b^+^ cells) and apoptotic cells within the liver were also observed (Fig. 7g, Supplementary Fig. 10)^37^. These combined effects were therefore sufficient to substantially increase the level of the transgene expression and extend the duration.

## Discussion

The translation of LNPs as a carrier for gene delivery has progressed tremendously over the past couple of years due to the success of COVID-19 mRNA vaccines. The biosafety and translatability of the LNPs have been demonstrated, making it extremely attractive for the field of gene therapy. Extending this to other therapeutic areas, however, requires systematic screening and optimization of the LNP formulation based on the requirements for specific target cell and tissue types, expression duration, etc. Both the choice of components and their molar ratios can drastically influence the nucleic acid encapsulation efficiency, stability of LNPs, cellular uptake, endosomal escape, and the release profile of the payload^8,9^. More importantly, screening for in vivo targets represents a greater challenge due to the throughput limitation. In developing this multi-step composition screening process, we successfully identified the top performing LNP formulations from the designed library consisting of 1080 formulations for liver-targeted transfection through i.v. administration. This designed screening process considers both in vitro and in vivo steps, which allows for rapid identification of effective formulations through a programmatic approach, and drastically reduces animal usage. This process can easily be translated to the development of delivery systems for other nucleic acid payloads including mRNA, siRNA, and miRNA, as well as alternative administrative routes such as oral, subcutaneous, and pulmonary delivery.

Recent literature indicates that LNPs with a particle size larger than 500 nm may yield a lower level of transgene expression in the liver after intravenous injection^14^. Therefore, this suggests that physical properties of LNPs, such as particle size range and surface charge, may be considered as key screening factors to establish a down-selection workflow for in vivo gene delivery.

On the other hand, there is insufficient evidence yet to identify the exact particle size as the determinant factor to rule out particles with a specific size limit as the first screening criterion. Based on these considerations, we did not put a limiting range for particle size or any of the other physical features, as the first set of factors for our screening workflow. We primarily relied on the performance factors, i.e., transfection efficiency at the cellular and tissue levels, along the various steps of the screening process, as the down selection criteria. In future work when sufficient data sets emerge to allow the identification of specific ranges of particle size and surface charge, an alternative screening workflow may be established to further reduce the number of particles tested in animal experiments. Overall, the advantage of the cluster-mode screening platform is that it reduces the number of animals, quantity of material, and time needed to identify lipid nanoparticle formulations capable of high transfection efficiency among a diverse, multi-dimensional library. Unlike some other screening strategies for gene delivery vehicles, this approach permits selection/grouping of formulations with a higher degree of flexibility based on in vitro and in vivo results in a sequential manner. For example, this study showcased an example of hepatocyte transfection, intrahepatic injection, and intravenous administration to down select the formulations from 1080 to top four. This sequence reflects selections at different physiological barriers including cellular delivery, tissue level, and organ targeting. The number of formulations per cluster can be adjusted based on the expression level and detection limit of the transgene. Compared with bar-coding screening approach which typically requires modification of cargos and assembling particles individually^31,32^, and relies on sophisticated single cell analysis, the method reported here provides a more simple and streamlined approach to identify effective formulations in various tissues and organ types.

Previous reports on LNP-enabled gene delivery systems argue that LNP composition influences tissue-targeting and transfection^7–10^. We revealed that the preferentially high transfection efficiency in the liver mediated by these selected LNP formulations is not directly related to in vivo biodistribution nor cellular uptake efficiency. Rather, the intracellular trafficking steps including endolysosomal escape and pDNA release play a more critical role. It is entirely possible for LNPs with similar characteristics and similar distribution among different organs and tissues to give different tissue-specific transfection outcomes and/or yield different transfection levels across different cell types. This is most clearly seen in the direct comparisons between top-performing formulations (DI-3, DI-8, FIII-7 and FIII-8) and selected formulations with less efficacy but similar characteristics (DI-6 and FIII-1). These findings further highlight the need for a rationale, multi-step screening approach when evaluating LNP formulations for each specific target organ and gene medicine application.

For therapeutic gene delivery, pDNA as a therapeutic payload offers unique advantages including more persistent transgene expression, higher stability, and a lower production cost, compared with the mRNA cargo. Our result also showed that optimized pDNA LNPs yielded 4 to 5-days of sustained transgene expression as opposed to the mRNA LNPs, which showed a sharp drop over two days. The innate immune activation against pDNA has been reported to induce gene silencing and inflammation response. Previously, CpG-depletion in pDNA sequence has been explored to address this issue. Here we demonstrated that a new approach via co-delivery of anti-inflammatory siRNAs with pDNA in the same LNP formulation that can effectively extend the transgene expression without relying on pDNA sequence modification. The inclusion of anti-inflammatory siRNAs reduced the recruitment of immune cells and the number of apoptotic cells after treatment with LNPs. Even with moderate reduction of STAT and NF-κB2 levels, this approach yields substantial improvement in the overall level and duration of the transgene in the liver. This strategy requires no sequence modification or complex delivery vehicles and can be easily adopted for other delivery systems and applications.

Overall, we report a multi-step composition screening platform that allowed us to programmatically identify the best-performing pDNA LNPs for liver-specific transgene expression from an LNP library of over 1000 formulations. This platform combines in vitro and in vivo screening strategies; it can be extended to other carrier systems and potentially for various administration routes. In addition, we revealed that the preferential transfection in the liver over other organs/tissues of the selected LNPs is not directly related to targeted in vivo distribution or cellular uptake efficiency of LNPs; rather the intracellular trafficking events including lysosome escape, DNA release, etc. play a more critical role. We deduced that LNPs with similar physical characteristics are distributed among different organs in a similar manner; but they show tissue-specific differences in transfection across different cell types due to differences in intracellular trafficking efficiency in a composition-dependent manner. Finally, we developed an innovative strategy that co-delivers anti-inflammatory siRNA and pDNA to further extend the expression of pDNA therapy. This LNP-based co-delivery strategy further highlights the unique advantages of an extended transgene expression profile using pDNA delivery and offers new opportunities for pDNA-based gene medicine applications.

## Methods

### Materials

DLin-MC3-DMA was purchased from MedKoo Biosciences. DSPC, DOPE, DOTAP, DDAB, 18PG (sodium salt) and 14PA (sodium salt) were purchased from Avanti Polar Lipids. Cholesterol was purchased from Sigma. DMG-PEG (MW 2000) (DMG-PEG2000) was purchased from NOF America Corporation. HepG2 cells (HB-8065^™^) and B16F10 cells (CRL-6475) were purchased from (American Type Culture Collection, USA). Reporter lysis buffer and luciferin assay solution were purchased from Promega. All pDNA was purchased from Aldevron. All siRNA was purchased from ThermoFisher.(STAT1 siRNA (Cat# AM16708), NFkβ2 siRNA (Cat# AM16708)) d-Luciferin (sodium salt) was purchased from Gold Biotechnology.

### Cell culture and high-throughput screening for transfection studies

For monolayer culture studies, HepG2 cells (American Type Culture Collection, USA) were seeded into 96-well plates at a cell density of 10,000 cells per well one day prior to transfection. The particles prepared were pipetted into EMEM medium at a final particle concentration of 1 μg pDNA/mL. For example, 8 μL of a particle suspension at 25 μg pDNA/mL was pipetted into the 200 μL culture media in the wells. A 24 h incubation was followed to allow transgene expression. When characterizing luciferase as the reporter, cells were lysed by reporter lysis buffer (Promega) using two freeze-thaw cycles, with the lysate characterized by a luminometer upon addition of luciferin assay solution (Promega) against a standard curve generated using luciferase samples (Promega).

### LNP synthesis and characterization

An organic phase was prepared by solubilizing with ethanol, a mixture of the helper lipid (DOTAP, DDAB, DOPE, DSPC, 14PA, 18PG) (Avanti), cholesterol (Sigma-Aldrich), DMG-PEG2000 (Avanti) and Dlin-MC3 DMA at a predetermined molar ratio. The aqueous phase was prepared in 25 mm magnesium acetate buffer (pH 4.0, Fisher) with Luc pDNA (firefly mLuc, Translate), mCherry pDNA, Cre pDNA or Cy5-labeled pDNA. All pDNAs were stored at −20 °C and were allowed to thaw on ice before use. For high-throughput screening, LNPs were prepared in a 96-well plate or 1.5 mL microcentrifuge tubes by directly adding ethanol phase to aqueous phase. For in vitro screening, LNPs were directly incubated with cells without dialysis. For in vivo batch analysis screening, LNPs in each cluster were mixed and dialyzed against DI water before injection into mice. For larger scale LNP production, the ethanol and aqueous phases were mixed at a 3:1 ratio in an FNC device using syringe pumps as previously described. Resultant LNPs were dialyzed against DI water in a 100,000 MWCO cassette (Fisher) at 4 °C for 24 h and were stored at 4 °C before injection. For cellular uptake studies in vitro, the LNPs were incubated with serum with volume ratio 2:1 (LNP/serum) for 30 min in 37 °C. The size, polydispersity index and zeta potentials of LNPs were measured using dynamic light scattering (ZetaPALS, Brookhaven Instruments). Diameters are reported as the intensity mean average.

### Animals and primary cells

All animal procedures were performed with ethical compliance and approval by the Johns Hopkins Institutional Animal Care and Use Committee (protocol #MO20E63). Female BALB/c mice (6–8 weeks) were obtained from the Jackson Laboratory and Ai9 mice (male, 6–8 weeks) bred in Johns Hopkins Animal Facilities and randomly grouped. Mice were generally fed a diet containing low fiber (5%), protein (20%) and fat (5–10%). The pelleted feed was supplied. Mice were supplied feed free choice and they ate 4–5 g a day (12 g/100 g body weight/day). Water was supplied free choice and they usually drank 3–5 ml a day (1.5 ml/10 g body weight/day). Water were supplied using automatic waterers. Mouse rooms were maintained at 30–70% relative humidity and a temperature of 18–26 °C (64–79 °F) with at least ten room air changes per hour. The mice were housed in standard shoebox cages with filter tops. Mice were provided with corncob as bedding.

The LNPs were injected i.v. via mouse lateral tail vein or intrahepatically via a small incision under sternum at a predetermined dose per mouse. The LNP suspensions were concentrated to 200 μg/mL of pDNA by an Amicon Ultra-2 centrifugal filter unit with a MWCO of 100 kDa. The mice were injected intraperitoneally with 100 μL of 30 mg/mL d-luciferin solution and were anesthetized in a ventilated anesthesia chamber with 1.5% isoflurane in oxygen and imaged at 5 min after the injection with an in vivo imaging system (IVIS, PerkinElmer). Luminescence was quantified using the Living Image software (PerkinElmer). For experiments in Ai9 mice, the Cre pDNA LNP formulations were prepared as described above and administered via i.v. injections at a pDNA dose of 25 μg per mouse (*n* = 2). After three days, mice were sacrificed, and the major organs were imaged using IVIS. For first 24 h of post-operative acre, after the operation, the animals were monitored at 30 min, 2 h, 4 h to ensure that they were not harmed. For animal welfare monitoring, after dosing or surgeries, the animals were monitored at 24 h and 48 h to ensure that they were not harmed. During surgery, heat support was provided. After surgery, 0.08 mL of buprenorphine (0.015 mg/mL) was given every 12 h for analgesia. For animal euthanasia, mice were euthanized by CO_2_ asphyxiation. The death of animal was verified by cervical dislocation. For in vitro transfection in primary hepatocytes, cells were isolated by using Hepatocyte Isolation System (Tissue Dissociation/Cell Isolation), BioAssay^™^ Kit (Cat. H2006-02) following manufacturer’s protocols. The hepatocytes were cultured in RPMI1640 medium supplemented with 10% fetal bovine serum at 37 °C in 5% CO_2_.

### Cell isolation and staining for flow cytometry

To quantify the mCherry+ or tdTom+ cells among different cell types in each organ, cell isolation and staining was performed, followed by flow cytometry analysis. For hepatocyte isolation, a two-step collagenase perfusion was executed. In detail, mice were anesthetized using isoflurane then fixed. Perfusion, initially using liver perfusion medium (Thermo Fisher) for 7–10 min, then switching to liver digestion medium (Thermo Fisher) for another 7–10 min, was performed. The liver was collected on a plate containing 10 mL of liver digestion medium and cut to release the hepatocytes. The released hepatocytes were then collected and washed with ice-cold hepatocyte wash medium (Thermo Fisher) and centrifuged at 50 × *g* for 5 min. The supernatant was decanted, and the pellet was resuspended with an ice-cold hepatocyte wash medium. The cell suspension was passed through a 100 μm filter. The hepatocyte suspension was washed twice with ice-cold hepatocyte wash medium and once with PBS via centrifugation (50 × *g*) for 5 min. Afterwards, the hepatocytes were further strained through a 100 μm filter and centrifuged at 50 × *g* for 5 min, and cells were resuspended in 500 µL of staining buffer. The antibodies used here were Brilliant Violet 605 anti-mouse CD45 (Biolegend #103140), Cyanine 5 anti-mouse CD326 (Biolegend #118214), APC/Cyanine7 anti-mouse CD31 (BioLegend #102440), PerCP-Cyanine 5.5 anti-mouse CD11b (BioLegend # 101228), and FITC anti-mouse CD11c (BioLegend #117306). The dilution ratio for all antibodies listed above was 1:200 with staining buffer (ThermoFisher #00422226). Flow data were acquired on SH800 and analyzed using FlowJo software.

For isolation and staining of spleen cells, the removed spleen was minced using a sterile blade and homogenized in 250 μL of digestion medium (45 units/μL collagenase I, 25 units/μL DNase I and 30 units/μL hyaluronidase). The spleen solution was transferred into a 15-mL tube that contained 5–10 mL of digestion medium. Next, the spleen solution was filtered using a 70 μm filter and washed once with PBS. Cells was pelleted at 300 × *g* for 5 min at 4 °C, and resuspended in 2 mL of red blood cell lysis buffer (BioLegend) and incubated on ice for 5 min. After incubation, 4 mL of cell staining buffer (BioLegend) was added and centrifuged again at 300 × *g* for 5 min. Cell pellet was washed with staining buffer for 3 times and stained with antibodies (total volume 100 μL) for 20 min in the dark at 4 °C. The stained cells were washed twice with 1 mL of PBS, then resuspended in 500 μL PBS for flow cytometry analysis. The antibodies used include Brilliant Violet 605 anti-mouse CD45 (BioLegend #103140), PerCP-Cyanine 5.5 anti-mouse CD11b (BioLegend #101228), APC anti-mouse CD11c (BioLegend #117309), FITC anti-mouse CD3 (BioLegend #100203) and PE-Cyanine 7 anti-mouse CD19 (BioLegend #115519).

For isolation and staining of lung cells, isolated lungs were minced using a sterile blade and then transferred into a 15 mL tube that contained 10 mL of 2× digestion medium (90 units/μL collagenase I, 50 units/μL DNase I, and 60 units/μL hyaluronidase) and incubated at 37 °C for 1 h with shaking. After incubation, any remaining lung tissue was homogenized. The following steps were similar to the spleen protocol described above. The antibodies used here were the same to that of hepatocytes.

### Quantitative endosomal escape assessments by Cellomics analysis

B16F10 cells expressing GFP-coupled galectin-8 (GFP-Gal8) were obtained by transfection using plasmids encoding Piggybac-transferase (Hera BioLabs) and Piggybac-transposon-GFP-Gal8 (Addgene) and a poly(β-amino ester) (PBAE) carrier^33^, then sorted by an SH800 cell sorter (Sony) twice. The cells were cultured in DMEM supplemented with 10% FBS at 100,000 cells per well. The particles were dosed at 24 h later as described above. After incubation for predetermined times, cells were washed with PBS for three times, fixed with 4% paraformaldehyde (PFA) solution, stained with Hoechst 33342, and then washed with PBS for three times. The plates were analyzed by a CellInsight CX7 High-content Analysis (HCA) platform (Thermo Fisher Scientific)^19^. Imaging was conducted at 20× magnification with a resolution of 1104 × 1104 pixel^2^ per field correlating with an area of 501.2 × 501.2 μm^2^. A total of 30 fields were analyzed inside each well of the plates; and the well-averaged results were generated by averaging all the cells in all the fields in each well.

### Statistical analysis

A two-tailed Student’s *t*-test or a one-way analysis of variance (ANOVA) was performed when comparing two groups or more than two groups, respectively. Statistical analysis was performed using Microsoft Excel (16.61.1) and Prism 8.0 (GraphPad). A difference is considered significant if *P* < 0.05, *P < 0.05, **P < 0.01, ***P < 0.001, ****P < 0.0001).

### Reporting summary

Further information on research design is available in the Nature Research Reporting Summary linked to this article.

## Supplementary information

Supplementary Information

Chemdraw files

Reporting Summary

## Data Availability

All data needed to evaluate the conclusions in the paper are present in the paper and/or the Supplementary Materials. Source data are provided with this paper.

## Source data

Source Data
