# Supplementary material for: Multi-step screening of DNA/lipid nanoparticles and co-delivery with siRNA to enhance and prolong gene expression
Source: Nat Commun. 2022 Jul 25;13:4282. doi: 10.1038/s41467-022-31993-y (PMC9310361; doi:10.1038/s41467-022-31993-y)
Supplement: Supplementary file 1 — Supplementary Information [file 41467_2022_31993_MOESM1_ESM.pdf]

Supporting Information

Multi-step Screening of DNA/Lipid Nanoparticles and Co-delivery with siRNA to Enhance and Prolong Gene Expression

Yining Zhu[1,2,3], Ruochen Shen[1,2,3], Ivan Vuong[1,2,3], Rebekah A. Reynolds[4,5], Melanie J. Shears[4,5], Zhi-Cheng Yao[2,3,6], Yizong Hu[1,2,3], Won June Cho[2,7], Jiayuan Kong[1,2,3], Sashank K. Reddy[1,2,8], Sean C. Murphy[4,5,9,10,*] and Hai-Quan Mao[1,2,3,6,*]

[1]Department of Biomedical Engineering, Johns Hopkins University School of Medicine, Baltimore, MD, USA.

[2]Institute for NanoBioTechnology, Johns Hopkins University, Baltimore, MD, USA.

[3]Translational Tissue Engineering Center, Johns Hopkins University School of Medicine, Baltimore, MD, USA.

[4]Department of Laboratory Medicine and Pathology, University of Washington, Seattle, WA, USA.

[5]Center for Emerging and Re-emerging Infectious Diseases, University of Washington, Seattle, WA, USA.

[6]Department of Materials Science and Engineering, Johns Hopkins University, Baltimore, MD, USA.

[7]Department of Chemical and Biomolecular Engineering, Johns Hopkins University, Baltimore, MD, USA.

[8]Department of Plastic and Reconstructive Surgery, Johns Hopkins University School of Medicine, Baltimore, MD, USA.

[9]Department of Microbiology, University of Washington, Seattle, WA, USA.

[10]Seattle Malaria Clinical Trials Center, Fred Hutch Cancer Research Center, Seattle, WA, USA.

*Corresponding author e-mail: hmao@jhu.edu; murphysc@uw.edu.

Supplementary Figures:

**Figure 1.**	Transfection efficiency of LNPs prepared using DDAB, DSPC or 14PA as the helper lipid and lipofectamine 3000.
**Figure 2.**	Effect of different composition parameters in formulations on size of LNPs
**Figure 3.**	Survival rate of mice for LNP-mediated pDNA delivery following i.v. injection via cluster-mode testing
**Figure 4.**	Survival rate of mice for LNP-mediated pDNA delivery following i.v. injection
**Figure 5.**	The tdTom expression levels in different organs mediated by LNP transfection in Ai9 mice at 3 days after a single i.v. administration
**Figure 6.**	Percentage of $tdTom^+$ cells in different organs mediated by LNP transfection in Ai9 mice at 3 days after a single i.v. administration
**Figure 7.**	Z-average size and zeta potential of LNPs within clusters DI and FIII
**Figure 8.**	Serum stability of the top-performing LNPs, DI-6 LNP and FIII-1 LNP
**Figure 9.**	Relative distribution of selected LNPs in the liver at 6, 12 and 24 h post i.v. injection
**Figure 10.**	Percent of apoptotic cells within the liver after i.v. injection of the selected LNPs
**Figure 11.**	Gating strategy example for flow cytometry data analysis

Supplementary Tables:

**Table 1.**	Formulation details and particle sizes for Top 32 LNPs with DOTAP as the helper lipid
**Table 2.**	Formulation details and particle sizes for Top 32 LNPs with DDAB as the helper lipid
**Table 3.**	Formulation details and particle sizes for Top 32 LNPs with DOPE as the helper lipid
**Table 4.**	Formulation details and particle sizes for Top 32 LNPs with DSPC as the helper lipid
**Table 5.**	Formulation details and particle sizes for Top 32 LNPs with 14PA as the helper lipid
**Table 6.**	Formulation details and particle sizes for Top 32 LNPs with 18PG as the helper lipid
**Table 7.**	Histogram details of particle sizes for Top 32 LNPs with different helper lipid

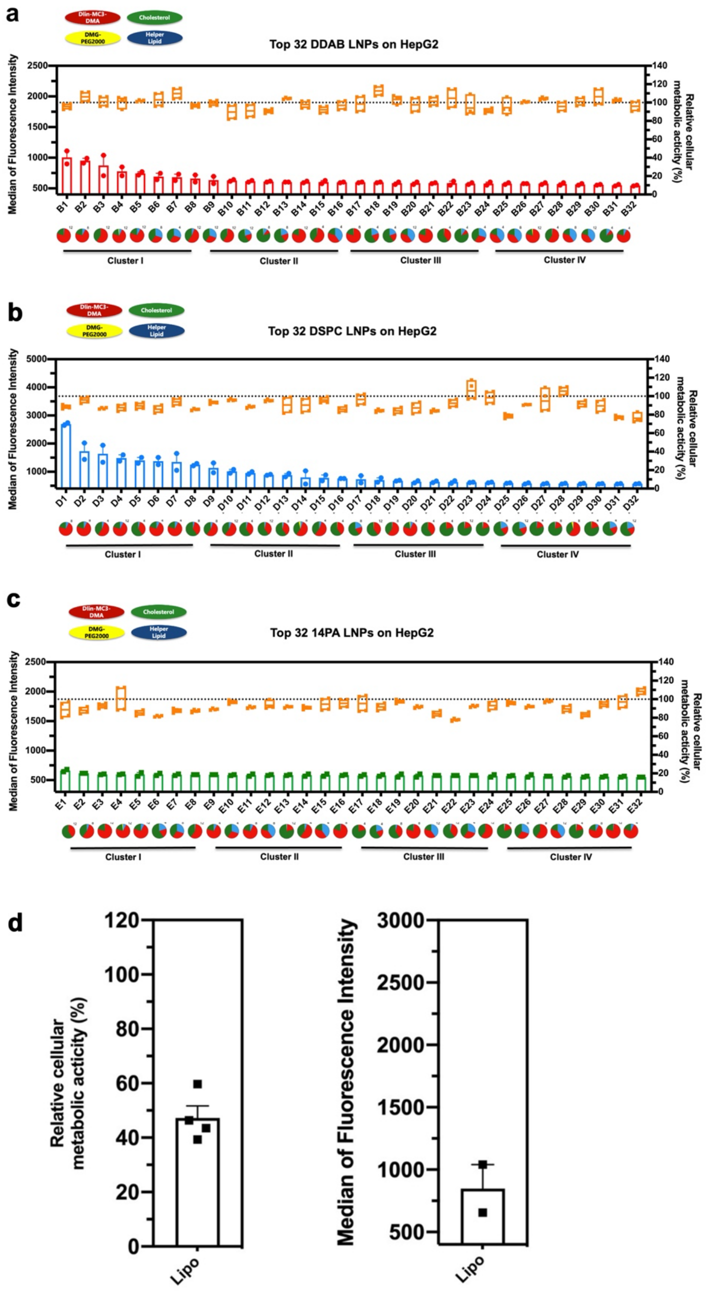

Supplementary Figure 1. The transfection efficiency of Groups B, D, and E LNPs prepared using DDAB, DSPC or 14PA as the helper lipid, respectively. FACS was used to further evaluate the transfection efficiency of (a) DDAB, (b) DSPC (c) 14PA LNPs and (d) lipofectamine 3000 (1 μg/mL pDNA (GFP), 72 h, n = 2). The efficiency of transgene expression of GFP as a reporter. Cellular metabolic activity was measured by alamarBlue assay (n = 4). Formulations were regrouped into four clusters, each containing eight formulations, based on their transgene expression level. Data are presented as mean ± S.D. The percentage of each component in the formulations is indicated by pie charts. Bars refer to the MFI (Median fluorescence intensity) value on the left, the boxes refer to the metabolic activity on the right. See Tables S1-S6 for molar percentages of all lipids used in the 32 formulations in each group of LNPs.

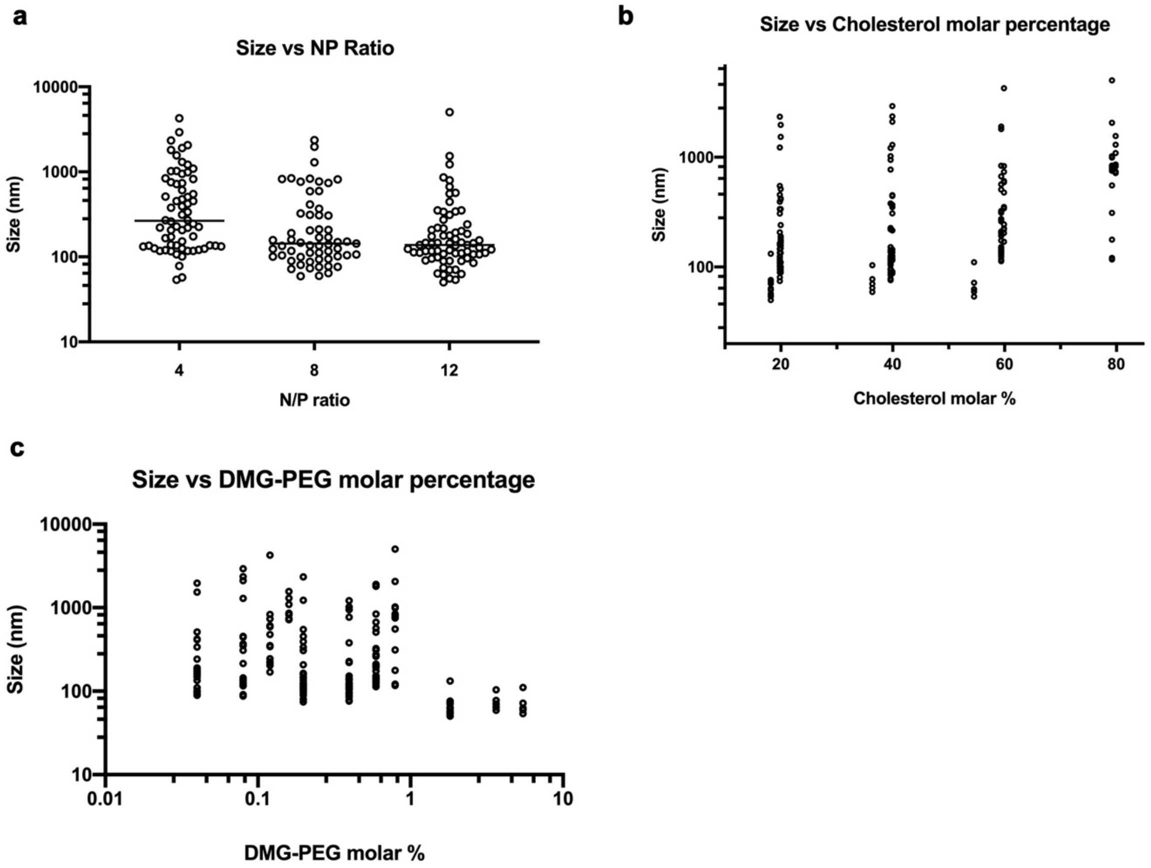

Supplementary Figure 2. Effect of various formulation parameters on the average size of LNPs. (a) Effect of NP ratio on the average size of LNPs. **(b)** Effect of cholesterol molar percentage on the average size of LNPs. **(c)** Effect of DMG-PEG molar percentage on the average size of LNPs. The average sizes and size distributions of the top performing LNPs (Top 32 formulations from each LNP group) were measured using dynamic light scattering (DLS).

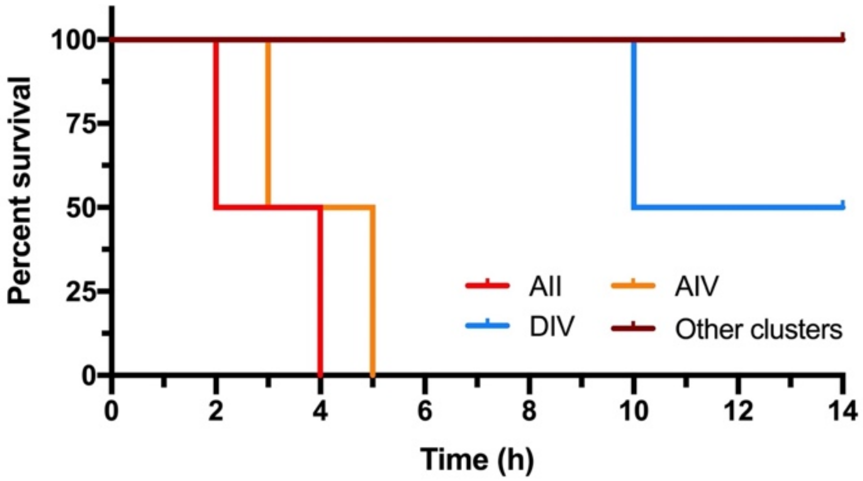

Supplementary Figure 3. Survival of Balb/c mice following a single intravenous injection of different clusters of LNP formulations. The three most toxic clusters (AII, AIV, DIV) were shown in this survival graph, where all other tested clusters did not cause animal death. LNPs were injected at a total pDNA dose of 100 µg (50% Luc + 50% mCherry) per mouse for each cluster (n = 2).

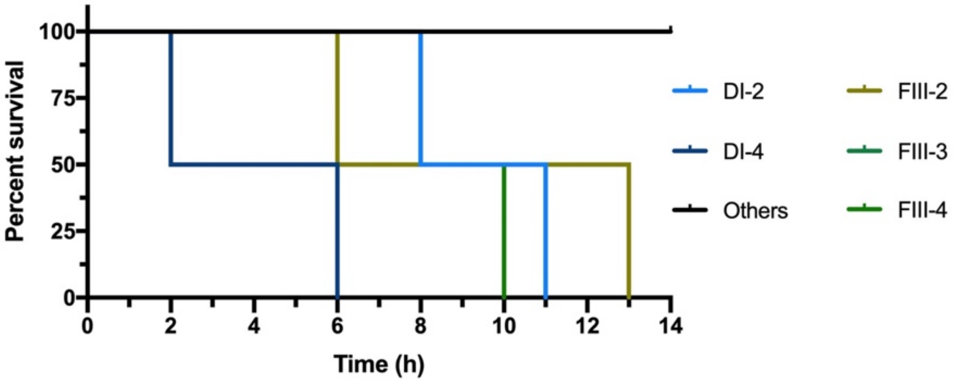

Supplementary Figure 4. Survival of Balb/c mice following a single intravenous injection of different LNP formulations. The five most toxic formulations (DI-2, DI-4, FIII-2, FIII-3, FIII-4) were shown in this survival graph, where all other tested formulations did not cause any death. LNPs were injected at a total pDNA dose of 50 µg (50% Luc + 50% mCherry) per mouse (n = 2).

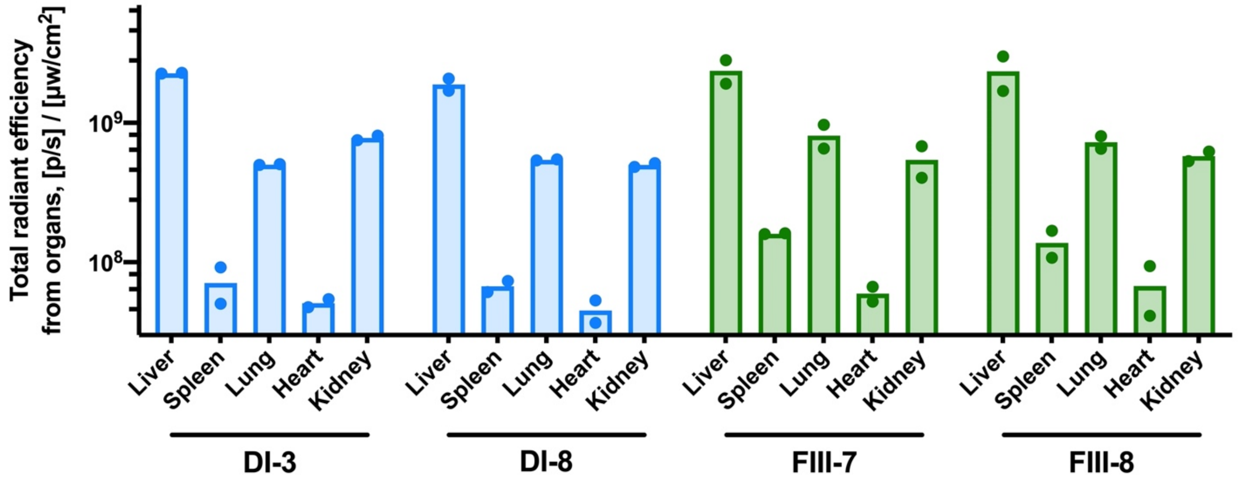

Supplementary Figure 5. Average tdTom expression levels in different organs at 3 days after a single i.v. injection of LNPs in Ai9 mice. The dose of Cre pDNA was 25 µg per mouse (i.v., n = 2). Data are presented as mean.

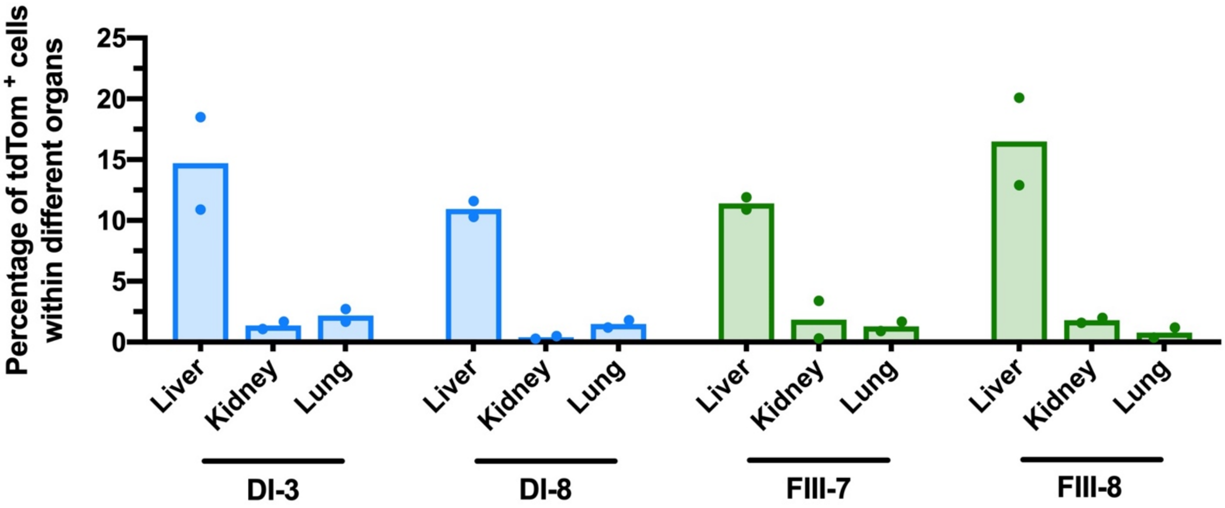

Supplementary Figure 6. Percentage of tdTom+ cells in the major organs at 3 days after a single i.v. injection of LNPs in Ai9 mice. FACS was used to quantify the percentage of tdTom+ cells in each organ (25 µg Cre pDNA per mouse, i.v., n = 2). Data are presented as mean.

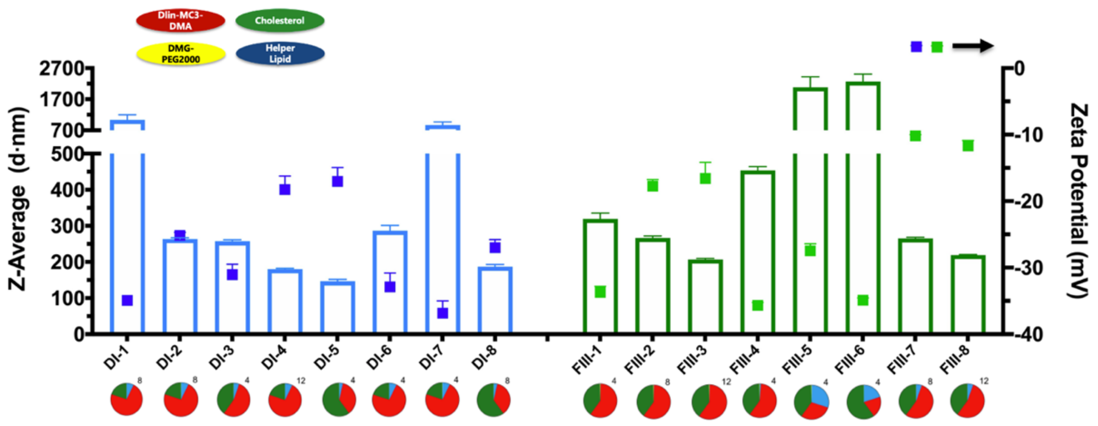

Supplementary Figure 7. Z-average size and zeta potential of different LNP formulations in clusters DI and FIII measured by DLS (n = 3). Data are presented as mean ± S.D. The percentage of each component in the formulations is indicated by pie charts. See Tables S4 and S6 for molar percentage used in the selected formulations.

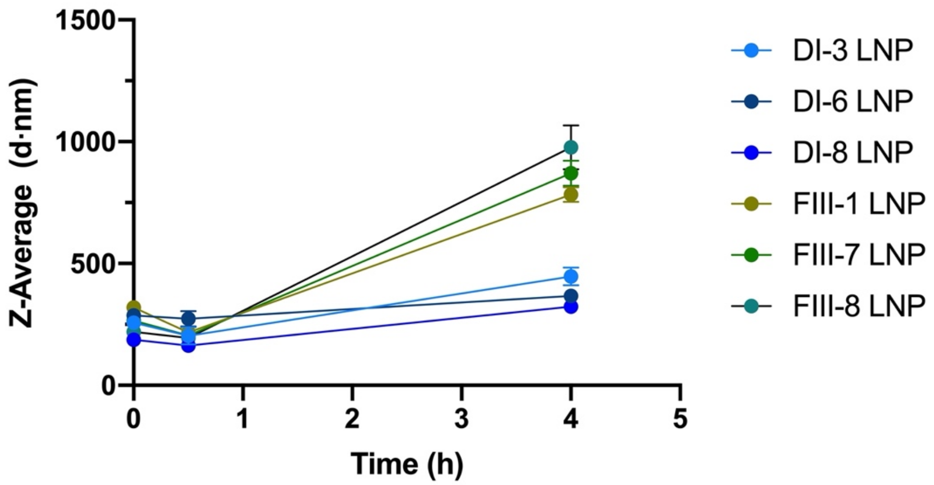

Supplementary Figure 8. Serum stability of the top-performing LNPs, DI-6 LNP and FIII-1 LNP measured by DLS (n = 3). Data are presented as mean values +/- SD. The LNP was added in PBS with 10% mouse serum and Z-average size was monitored by Zeta-sizer.

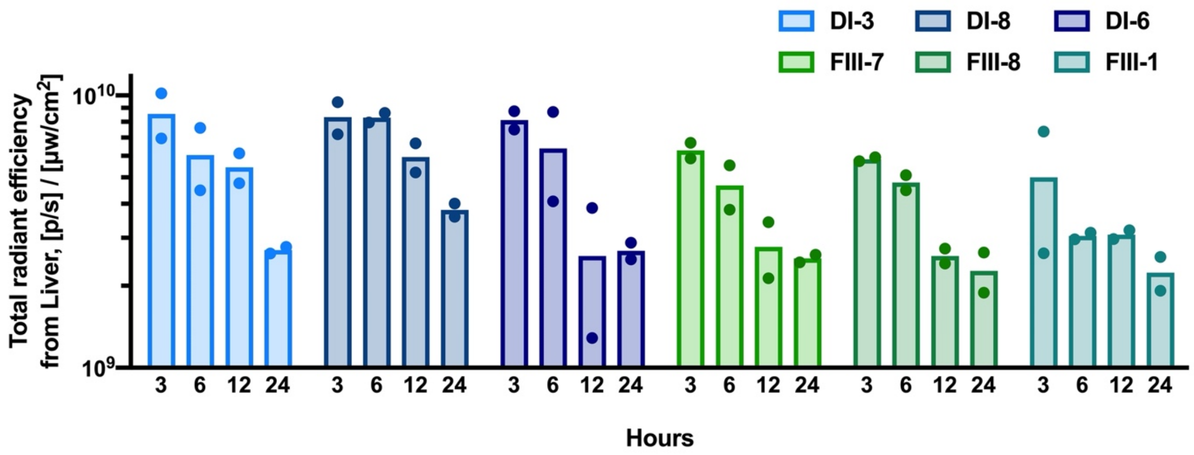

Supplementary Figure 9. Biodistribution in the liver at 6, 12 and 24 h post-injection of LNPs in different clusters (DI-3, DI-6, DI-8, FIII-1, FIII-7, and FIII-8). Biodistribution in the liver was determined at 6, 12 and 24 h post-injection of different clusters of LNPs at a total pDNA dose of 30 µg per mouse (85% Luc + 15% Cy5-labeled 1216), i.v., n = 2).

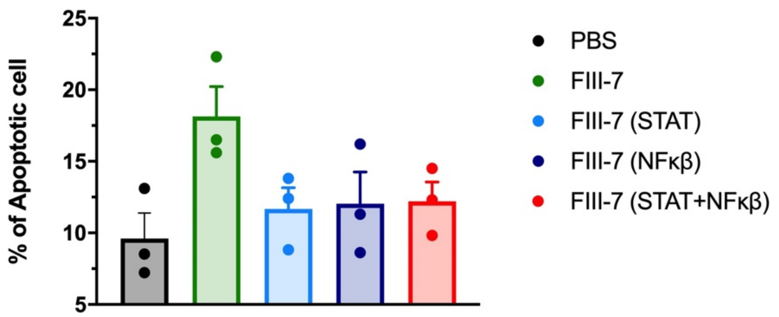

Supplementary Figure 10. Percent of apoptotic cells (Zombie Violet-Apotracker Green+ cells) within the liver after one single i.v. injection of the selected LNPs. FACS was used to quantify the percentage of Zombie Violet-Apotracker Green+ cells in the liver (25 µg Luc pDNA per mouse, 2.5 µg siRNA for each transcription factor per mouse, i.v., n = 3). Data are presented as mean ± S.E.M.

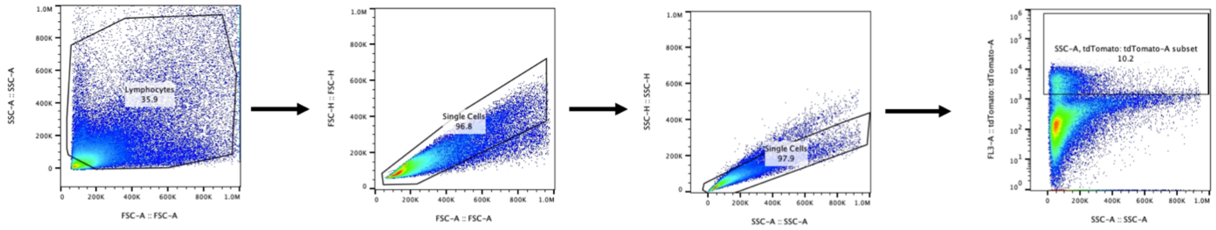

Supplementary Figure 11. Gating strategy example for flow cytometry data analysis. Gating was first based on FSC/SSC together with FSC-A/FSC-H and SSC-A/SSC-H (singlet populations). The cell populations within the gate were further analyzed based on expression of targetd protein. Single positive staining were used to determine the positive.

Supplementary Table 1. Formulation details and particle sizes for Top 32 LNPs in Group A with DOTAP as the helper lipid

Code	Mol %				N/P Ratio	Z-Average (nm)	PDI
	DOTAP	Dlin-MC3	Chol	DMG-PEG			
A1	5.45	54.55	39.60	0.40	4	718.77 ± 22.9	0.29 ± 0.28
A2	5.45	54.55	39.60	0.40	12	738.2 ± 32.9	0.2 ± 0.13
A3	20.00	20.00	59.88	0.12	8	737.03 ± 68.1	0.76 ± 0.22
A4	5.45	54.55	39.60	0.40	8	169.8 ± 8.9	0.73 ± 0.02
A5	40.00	40.00	19.96	0.04	12	87.51 ± 3.6	0.25 ± 0.02
A6	3.64	36.36	59.41	0.59	12	90.77 ± 0.7	0.28 ± 0.01
A7	30.00	30.00	39.92	0.08	8	89.37 ± 1.9	0.27 ± 0.03
A8	3.64	36.36	59.41	0.59	8	311.6 ± 71.3	1.0
A9	3.64	36.36	59.41	0.59	4	152.23 ± 11.1	0.55 ± 0.06
A10	10.00	10.00	79.20	0.80	8	146.8 ± 10.1	0.44 ± 0.09
A11	20.00	20.00	59.41	0.59	12	174.2 ± 7.0	0.45 ± 0.02
A12	30.00	30.00	39.60	0.40	8	116.57 ± 1.7	0.2 ± 0.01
A13	30.00	30.00	39.92	0.08	12	104.61 ± 7.0	0.43 ± 0.1
A14	40.00	40.00	19.80	0.20	8	137.73 ± 6.2	0.45 ± 0.01
A15	40.00	40.00	19.80	0.20	12	752.77 ± 140.2	0.23 ± 0.21
A16	30.00	30.00	39.60	0.40	12	765.23 ± 42.1	0.37 ± 0.04
A17	10.00	10.00	79.21	0.79	12	177.6 ± 2.1	0.8 ± 0.07
A18	20.00	20.00	59.88	0.12	4	260.67 ± 8.1	0.78 ± 0.22
A19	20.00	20.00	54.55	5.45	8	149.27 ± 0.9	0.44 ± 0.04
A20	40.00	40.00	19.80	0.20	4	124.1 ± 3.0	0.49 ± 0.08
A21	20.00	20.00	59.41	0.59	8	78.03 ± 0.4	0.2 ± 0.02
A22	10.00	10.00	79.84	0.16	4	75.96 ± 0.5	0.23 ± 0.01
A23	1.82	18.18	79.21	0.79	12	89.37 ± 1.0	0.21 ± 0.01
A24	20.00	20.00	54.55	5.45	4	101.27 ± 3.8	0.33 ± 0.03
A25	40.00	40.00	18.18	1.82	8	80.29 ± 2.1	0.25 ± 0.01
A26	30.00	30.00	39.60	0.40	4	74.51 ± 1.4	0.26 ± 0.03
A27	20.00	20.00	59.41	0.59	4	59.71 ± 0.6	0.21 ± 0.03
A28	40.00	40.00	18.18	1.82	12	62.7 ± 0.9	0.23 ± 0.02
A29	10.00	10.00	79.84	0.16	8	59.21 ± 0.9	0.2 ± 0.01
A30	20.00	20.00	54.55	5.45	12	73.46 ± 1.9	0.24 ± 0.03
A31	30.00	30.00	36.36	3.64	8	70.44 ± 1.8	0.25 ± 0.03
A32	10.00	10.00	79.20	0.80	4	53.88 ± 0.8	0.19 ± 0.02

Supplementary Table 2. Formulation details and particle sizes for Top 32 LNPs in Group B with DDAB as the helper lipid

Code	Mol %				N/P Ratio	Z-Average (nm)	PDI
	DDAB	Dlin-MC3	Chol	DMG-PEG			
B1	1.57	78.43	19.96	0.04	12	186.73 ± 7.1	0.34 ± 0.06
B2	7.27	72.73	19.96	0.04	8	144.03 ± 7.8	0.53 ± 0.05
B3	0.59	59.41	39.92	0.08	12	215.0 ± 87.0	0.66 ± 0.28
B4	7.27	72.73	18.18	1.82	12	110.17 ± 2.5	0.34 ± 0.03
B5	7.27	72.73	19.96	0.04	12	119.63 ± 2	0.34 ± 0.02
B6	30.00	30.00	39.60	0.40	8	100.76 ± 2.9	0.23 ± 0.02
B7	30.00	30.00	39.92	0.08	4	98.45 ± 1.1	0.28 ± 0.03
B8	5.45	54.55	39.92	0.80	12	1091.67 ± 116.2	0.34 ± 0.22
B9	30.00	30.00	39.60	0.40	12	476.1 ± 63.6	0.96 ± 0.06
B10	1.18	58.82	39.92	0.08	12	125.93 ± 3.1	0.18 ± 0.04
B11	20.00	20.00	59.41	0.59	12	165.53 ± 1.4	0.23 ± 0.02
B12	10.00	10.00	79.21	0.79	8	99.51 ± 1.3	0.17 ± 0.01
B13	20.00	20.00	59.41	0.59	8	89.13 ± 2.7	0.17 ± 0.01
B14	0.40	79.60	19.96	0.04	12	135.2 ± 3.1	0.2 ± 0.03
B15	5.45	54.55	39.60	0.40	4	101.6 ± 0.5	0.25 ± 0.02
B16	40.00	40.00	19.96	0.04	4	837.53 ± 277.7	1 ± 0
B17	0.40	79.60	19.80	0.20	8	945.37 ± 290	0.92 ± 0.14
B18	20.00	20.00	59.41	0.59	4	117.5 ± 5.2	0.18 ± 0.02
B19	20.00	20.00	59.88	0.12	4	336.67 ± 20.7	0.3 ± 0.05
B20	40.00	40.00	19.96	0.04	12	991.9 ± 189.9	0.3 ± 0.22
B21	0.40	79.60	19.80	0.20	4	813.77 ± 67.6	0.25 ± 0.13
B22	0.78	39.22	59.41	0.59	4	143.67 ± 8.1	0.52 ± 0.07
B23	10.00	10.00	79.21	0.79	4	129.03 ± 1.9	0.52 ± 0.11
B24	30.00	30.00	39.60	0.40	4	136.27 ± 0.6	0.17 ± 0
B25	40.00	40.00	19.80	0.20	4	111.87 ± 0.9	0.22 ± 0.01
B26	40.00	40.00	19.80	0.20	8	84.67 ± 0.6	0.17 ± 0.06
B27	0.40	79.60	18.18	1.82	12	163.83 ± 10	0.27 ± 0.04
B28	1.18	58.82	39.60	0.40	4	116.3 ± 2.2	0.2 ± 0.02
B29	40.00	40.00	19.96	0.04	8	95.38 ± 1.3	0.12 ± 0.06
B30	40.00	40.00	19.80	0.20	12	132.6 ± 6.6	0.39 ± 0.02
B31	10.00	10.00	79.84	0.16	4	61.63 ± 1.5	0.19 ± 0.02
B32	7.27	72.73	19.80	0.20	4	55.53 ± 0.9	0.15 ± 0.05

Supplementary Table 3. Formulation details and particle sizes for Top 32 LNPs in Group C with DOPE as the helper lipid

Code	Mol %				N/P Ratio	Z-Average (nm)	PDI
	DOPE	Dlin-MC3	Chol	DMG-PEG			
C1	30.00	30.00	39.92	0.08	12	156.73 ± 3.3	0.44 ± 0.13
C2	30.00	30.00	39.92	0.08	8	452.1 ± 62.4	0.52 ± 0.17
C3	5.45	54.55	39.60	0.40	12	308.1 ± 79.9	0.7 ± 0.26
C4	40.00	40.00	19.96	0.04	8	351.57 ± 24.8	0.42 ± 0.09
C5	7.27	72.73	19.96	0.04	12	420.57 ± 61.3	1.0
C6	7.27	72.73	19.80	0.20	12	413.63 ± 112.3	0.71 ± 0.25
C7	40.00	40.00	19.96	0.04	12	241.47 ± 16.1	0.42 ± 0.01
C8	30.00	30.00	39.60	0.40	12	147.83 ± 8.7	0.39 ± 0.05
C9	30.00	30.00	39.60	0.40	8	122.37 ± 1.9	0.35 ± 0.04
C10	30.00	30.00	39.60	0.40	4	107.27 ± 3.8	0.36 ± 0.08
C11	1.57	78.43	19.80	0.20	12	116.8 ± 4.5	0.43 ± 0.02
C12	1.57	78.43	19.96	0.04	12	273.77 ± 11	0.47 ± 0.02
C13	0.79	79.21	19.80	0.20	12	111.43 ± 4.1	0.35 ± 0.03
C14	40.00	40.00	19.80	0.20	8	118.17 ± 5.4	0.47 ± 0.04
C15	20.00	20.00	59.41	0.60	4	2056.67 ± 415.9	0.6 ± 0.22
C16	7.27	72.73	18.18	1.82	12	794.13 ± 106.4	0.8 ± 0.21
C17	40.00	40.00	18.18	1.82	4	1902 ± 305.8	0.55 ± 0.03
C18	40.00	40.00	19.80	0.20	8	136.5 ± 5.5	0.31 ± 0.03
C19	0.78	39.22	59.41	0.59	12	116.73 ± 7.3	0.21 ± 0.04
C20	40.00	40.00	19.80	0.20	4	221.3 ± 27.8	0.35 ± 0.12
C21	10.00	10.00	79.21	0.79	12	111.63 ± 1.4	0.11 ± 0.01
C22	30.00	30.00	39.92	0.08	8	220.17 ± 8.4	0.43 ± 0.04
C23	0.40	79.60	19.80	0.20	12	2339 ± 786.7	0.6 ± 0.11
C24	20.00	20.00	59.41	0.59	4	158.9 ± 3.8	0.25 ± 0.03
C25	20.00	20.00	59.41	0.59	12	161.83 ± 2.7	0.36 ± 0.02
C26	40.00	40.00	19.96	0.04	4	53.74 ± 2.1	0.18 ± 0.03
C27	10.00	10.00	79.21	0.79	4	110.33 ± 2	0.44 ± 0.01
C28	40.00	40.00	18.18	1.82	8	70.34 ± 0.7	0.19 ± 0.05
C29	30.00	30.00	36.36	3.64	12	131.83 ± 2.1	0.25 ± 0.03
C30	40.00	40.00	18.18	1.82	12	76.21 ± 0.9	0.37 ± 0.07
C31	30.00	30.00	36.36	3.64	8	50.12 ± 0.7	0.22 ± 0.02
C32	20.00	20.00	54.55	5.45	12	103.97 ± 1.6	0.21 ± 0.03

Supplementary Table 4. Formulation details and particle sizes for Top 32 LNPs in Group D with DSPC as the helper lipid

Code	Mol %				N/P Ratio	Z-Average (nm)	PDI
	DSPC	Dlin-MC3	Chol	DMG-PEG			
D1	7.27	72.73	19.96	0.04	8	1561 ± 10.1	0.71 ± 0.25
D2	7.27	72.73	19.80	0.20	8	244.63 ±1 2.3	0.39 ± 0.03
D3	5.45	54.55	39.92	0.08	4	257.36 ± 4.25	0.28 ± 0.15
D4	7.27	72.73	19.96	0.04	12	2921 ± 146.3	0.23 ± 0.22
D5	3.64	36.36	59.88	0.12	4	171.57 ± 2.7	0.35 ± 0.05
D6	7.27	72.73	19.96	0.04	4	168.73 ± 2.9	0.32 ± 0.02
D7	7.27	72.73	19.80	0.20	4	178.3 ± 2.2	0.33 ± 0.06
D8	3.64	36.36	59.88	0.12	8	186.67 ± 6.67	0.13 ± 0.08
D9	5.45	54.55	39.60	0.40	8	342 ± 30.3	0.78 ± 0.2
D10	5.45	54.55	39.60	0.40	12	821.4 ± 197	0.98 ± 0.03
D11	3.64	36.36	59.41	0.59	4	1020.8 ± 181.1	0.26 ± 0.15
D12	3.64	36.36	59.41	0.59	12	312.7 ± 57.8	0.83 ± 0.02
D13	3.64	36.36	59.41	0.59	8	192.37 ± 24.4	0.75 ± 0.07
D14	5.45	54.55	36.36	3.64	8	135.03 ± 8.2	0.48 ± 0.05
D15	5.45	54.55	39.60	0.40	4	106.43 ± 4.5	0.37 ± 0.03
D16	0.78	39.22	59.41	0.59	4	117.3 ± 7	0.47 ± 0.09
D17	20.00	20.00	59.88	0.12	8	839.77 ± 8.3	0.17 ± 0.08
D18	0.40	39.60	59.41	0.59	12	553.2 ± 46.6	0.65 ± 0.08
D19	1.18	58.82	39.60	0.40	8	507.5 ± 525.3	0.92 ± 0.14
D20	7.27	72.73	18.18	1.82	4	323.9 ± 69.1	1 ± 0
D21	0.40	39.60	59.41	0.59	4	111.2 ± 58.6	0.52 ± 0.12
D22	0.10	19.90	79.21	0.79	4	1213.03 ± 1334	0.79 ± 0.35
D23	1.82	18.18	79.21	0.79	12	125.37 ± 6.7	0.3 ± 0.06
D24	1.82	18.18	79.21	0.79	4	111.67 ± 1.7	0.19 ± 0.01
D25	20.00	20.00	59.41	0.59	8	125.03 ± 1.9	0.24 ± 0.01
D26	20.00	20.00	59.88	0.12	12	89.54 ± 4.8	0.24 ± 0.03
D27	1.82	18.18	79.84	0.16	4	1804 ± 196.7	0.27 ± 0.07
D28	1.82	18.18	79.21	0.79	8	210.37 ± 14.8	0.35 ± 0.03
D29	1.18	58.82	36.36	3.64	8	668.47 ± 61.7	0.86 ± 0.14
D30	0.20	19.80	79.21	0.79	4	57.14 ± 1.2	0.2 ± 0.09
D31	20.00	20.00	59.41	0.59	4	77.43 ± 19.2	0.47 ± 0.13
D32	20.00	20.00	59.41	0.59	12	64.37 ± 1	0.16 ± 0.01

Supplementary Table 5. Formulation details and particle sizes for Top 32 LNPs in Group E with 14PA as the helper lipid

Code	Mol %				N/P Ratio	Z-Average (nm)	PDI
	14PA	Dlin-MC3	Chol	DMG-PEG			
E1	0.20	39.80	59.88	0.12	12	1301.9 ± 535.2	0.45 ± 0.48
E2	5.45	54.55	39.60	0.40	8	4257.33 ± 3229	0.38 ± 0.42
E3	0.40	79.60	19.80	0.20	12	204.13 ± 18.7	0.43 ± 0.07
E4	7.27	72.73	18.18	1.82	12	352.4 ± 70.7	0.92 ± 0.03
E5	7.27	72.73	19.96	0.04	12	2107 ± 242.9	0.42 ± 0.14
E6	20.00	20.00	59.41	0.59	4	135.33 ± 3	0.28 ± 0.01
E7	30.00	30.00	39.92	0.08	8	1294.33 ± 409.5	0.77 ± 0.28
E8	1.18	58.82	39.60	0.40	12	443.63 ± 27.4	0.65 ± 0.17
E9	7.27	72.73	19.80	0.20	4	173.8 ± 13.2	0.46 ± 0.12
E10	30.00	30.00	39.92	0.08	4	157.47 ± 2.5	0.22 ± 0.03
E11	7.27	72.73	19.96	0.04	8	134.33 ± 14.9	0.5 ± 0.07
E12	40.00	40.00	19.96	0.04	8	93.69 ± 2.6	0.25 ± 0.03
E13	1.81	18.18	79.21	0.79	8	337.8 ± 16.3	0.59 ± 0.14
E14	5.45	54.55	39.60	0.40	4	1534.33 ± 421.6	0.78 ± 0.38
E15	40.00	40.00	19.80	0.20	4	121.1 ± 5.9	0.22 ± 0.04
E16	1.57	78.43	19.80	0.20	8	117.4 ± 4.4	0.26 ± 0.03
E17	0.10	19.90	79.84	0.16	4	5021.4 ± 4995.1	0.71 ± 0.38
E18	20.00	20.00	59.88	0.12	4	138.37 ± 3.2	0.27 ± 0.04
E19	3.64	36.36	59.88	0.12	8	113.1 ± 0.7	0.13 ± 0.05
E20	1.57	78.43	19.96	0.04	8	106.97 ± 2.8	0.27 ± 0.07
E21	40.00	40.00	19.96	0.04	12	124.4 ± 1.2	0.33 ± 0.05
E22	3.64	36.36	59.41	0.59	12	770.53 ± 268.1	1 ± 0
E23	30.00	30.00	39.60	0.40	4	94.43 ± 1.9	0.23 ± 0.05
E24	0.30	59.70	39.92	0.08	12	108.33 ± 5.3	0.3 ± 0.03
E25	1.82	18.18	79.21	0.79	8	547.33 ± 141.9	0.92 ± 0.13
E26	30.00	30.00	39.60	0.40	8	393.1 ± 189.3	0.95 ± 0.08
E27	0.30	59.70	39.92	0.08	8	206.97 ± 94	0.61 ± 0.17
E28	40.00	40.00	19.80	0.20	12	306.33 ± 34.6	0.76 ± 0.05
E29	1.82	18.18	79.21	0.79	4	145.87 ± 8.7	0.3 ± 0.04
E30	7.27	72.73	19.80	0.20	8	1226.67 ± 135.7	0.48 ± 0.15
E31	1.57	78.43	19.80	0.20	12	107.37 ± 0.6	0.3 ± 0.04
E32	7.27	72.73	19.96	0.04	4	63.76 ± 1.9	0.23 ± 0.03

Supplementary Table 6. Formulation details and particle sizes for Top 32 LNPs in Group F with 18PG as the helper lipid

Code	Mol %				N/P Ratio	Z-Average (nm)	PDI
	18PG	Dlin-MC3	Chol	DMG-PEG			
F1	0.20	39.80	59.41	0.59	4	823 ± 87.2	0.22 ± 0.07
F2	0.40	39.60	59.41	0.59	4	857.6 ± 7.9	0.22 ± 0.09
F3	0.20	39.80	59.41	0.59	8	225.13 ± 20.4	0.67 ± 0.13
F4	0.59	59.41	39.60	0.40	4	220 ± 17.2	0.65 ± 0.03
F5	5.45	54.55	39.60	0.40	12	607.53 ± 45	0.43 ± 0.04
F6	1.18	58.82	39.92	0.08	12	593.47 ± 8.80	0.97 ± 0.06
F7	0.20	39.80	54.55	5.45	8	367.43 ± 103	0.93 ± 0.12
F8	0.78	39.22	59.88	0.12	4	2356.6 ± 892	0.46 ± 0.16
F9	3.64	36.36	59.88	0.12	4	115.53 ± 2.9	0.45 ± 0.08
F10	0.20	39.80	59.88	0.12	8	135.9 ± 4.8	0.11 ± 0.09
F11	0.30	59.70	39.92	0.08	8	512.2 ± 122.9	0.88 ± 0.11
F12	7.27	72.73	19.96	0.04	8	190 ± 2.5	0.38 ± 0.04
F13	0.40	39.60	59.41	0.59	12	1969.5 ± 1727	0.98 ± 0.02
F14	3.64	36.36	59.41	0.59	12	269.74 ± 188.2	0.78 ± 0.27
F15	0.40	39.60	59.41	0.59	8	206.33 ± 66.1	0.76 ± 0.1
F16	0.20	39.80	59.41	0.59	12	312.13 ± 328.5	0.73 ± 0.24
F17	0.30	59.70	39.60	0.40	4	139.83 ± 34.7	0.56 ± 0.06
F18	0.30	59.70	39.60	0.40	8	207 ± 24.2	0.8 ± 0.13
F19	0.30	59.70	39.60	0.40	12	567.03 ± 229.5	0.99 ± 0.02
F20	1.18	58.82	39.60	0.40	4	201.43 ± 10.1	0.62 ± 0.08
F21	30.00	30.00	39.60	0.40	4	1021.3 ± 140	1 ± 0
F22	20.00	20.00	59.88	0.12	4	119.97 ± 2.8	0.21 ± 0.03
F23	5.45	54.55	39.92	0.08	8	265.46 ± 2.57	0.23 ± 0.02
F24	5.45	54.55	39.92	0.08	12	218.93 ± 1.59	0.16 ± 0.02
F25	1.82	18.18	79.84	0.16	8	137.57 ± 4.4	0.38 ± 0.06
F26	1.57	78.43	19.96	0.04	8	142.83 ± 1.7	0.45 ± 0.03
F27	0.59	59.41	39.60	0.40	12	96.27 ± 1.8	0.23 ± 0.02
F28	1.57	78.43	19.80	0.20	4	454 ± 62.5	0.6 ± 0.18
F29	7.27	72.73	19.80	0.20	8	123.97 ± 3.1	0.33 ± 0.04
F30	0.79	79.21	19.80	0.20	12	146.23 ± 3.7	0.38 ± 0.07
F31	1.57	78.43	19.96	0.04	4	226.93 ± 7.9	0.66 ± 0.06
F32	1.82	18.18	79.84	0.16	12	71.78 ± 0.9	0.37 ± 0.01

Supplementary Table 7. Histogram details of particle sizes for Top 32 LNPs with different helper lipid

BIN CENTER	NUMBER OF FORMULATION					
	DOTAP	DDAB	DOPE	DSPC	14PA	18PG
**50**	3	2	2	1	0	0
**75**	7	1	2	2	1	1
**100**	6	8	5	3	5	1
**125**	2	8	6	4	6	4
**150**	4	2	4	1	3	5
**175**	3	3	0	3	1	0
**200**	0	0	0	2	2	4
**225**	0	1	2	0	0	3
**250**	1	0	1	1	0	0
**275**	0	0	1	0	0	1
**300**	1	0	1	0	1	1
**325**	0	1	0	2	0	0
**350**	0	0	1	1	2	0
**375**	0	0	0	0	0	2
**400**	0	0	0	0	1	0
**500**	0	1	3	3	2	4
**700**	5	0	1	1	1	1
**>800**	0	5	3	8	7	5
