# Supplementary material for: Multi-step screening of DNA/lipid nanoparticles and co-delivery with siRNA to enhance and prolong gene expression
Source: Nat Commun. 2022 Jul 25;13:4282. doi: 10.1038/s41467-022-31993-y (PMC9310361; doi:10.1038/s41467-022-31993-y)
Supplement: Supplementary file 3 — Reporting Summary [file 41467_2022_31993_MOESM3_ESM.pdf]

nature portfolio

Corresponding author(s): Hai-Quan Mao, Sean C. Murphy

Last updated by author(s): Jun 14, 2022

Reporting Summary

Nature Portfolio wishes to improve the reproducibility of the work that we publish. This form provides structure for consistency and transparency in reporting. For further information on Nature Portfolio policies, see our Editorial Policies and the Editorial Policy Checklist.

Statistics

For all statistical analyses, confirm that the following items are present in the figure legend, table legend, main text, or Methods section.

n/a	Confirmed	
☐	☒	The exact sample size (*n*) for each experimental group/condition, given as a discrete number and unit of measurement
☐	☒	A statement on whether measurements were taken from distinct samples or whether the same sample was measured repeatedly
☐	☒	The statistical test(s) used AND whether they are one- or two-sided<br>*Only common tests should be described solely by name; describe more complex techniques in the Methods section.*
☒	☐	A description of all covariates tested
☐	☒	A description of any assumptions or corrections, such as tests of normality and adjustment for multiple comparisons
☐	☒	A full description of the statistical parameters including central tendency (e.g. means) or other basic estimates (e.g. regression coefficient) AND variation (e.g. standard deviation) or associated estimates of uncertainty (e.g. confidence intervals)
☐	☒	For null hypothesis testing, the test statistic (e.g. *F*, *t*, *r*) with confidence intervals, effect sizes, degrees of freedom and *P* value noted<br>*Give P values as exact values whenever suitable.*
☒	☐	For Bayesian analysis, information on the choice of priors and Markov chain Monte Carlo settings
☒	☐	For hierarchical and complex designs, identification of the appropriate level for tests and full reporting of outcomes
☒	☐	Estimates of effect sizes (e.g. Cohen's *d*, Pearson's *r*), indicating how they were calculated

Our web collection on statistics for biologists contains articles on many of the points above.

Software and code

Policy information about availability of computer code

Data collection	SH800S, Living Image Software (IVIS Imaging Systems) v4.5.5
Data analysis	Graphpad Prism v8, FlowJo v10, Living Image Software (IVIS Imaging Systems) v4.5.5, Microsoft Excel v16.61.1

For manuscripts utilizing custom algorithms or software that are central to the research but not yet described in published literature, software must be made available to editors and reviewers. We strongly encourage code deposition in a community repository (e.g. GitHub). See the Nature Portfolio guidelines for submitting code & software for further information.

Data

Policy information about availability of data

All manuscripts must include a data availability statement. This statement should provide the following information, where applicable:

- Accession codes, unique identifiers, or web links for publicly available datasets
- A description of any restrictions on data availability
- For clinical datasets or third party data, please ensure that the statement adheres to our policy

All data needed to evaluate the conclusions in the paper are present in the paper and/or the Supplementary Materials. Source data are provided with this paper.

Field-specific reporting

Life sciences study design

All studies must disclose on these points even when the disclosure is negative.

Sample size	No sample size calculation was performed. Sample sizes are clearly reported in the Figure Legend. For in vivo and in vitrohighthroughput screening experiments, sample sizes were determined as 2 for reducing animal usage, time and materials. Otherwise, the sample size were determined by allowable error size and accuracy, and resources (generally n>=3). The sample size of each experiment is in agreement with those studies already published and with the need for statistical analysis.
Data exclusions	No data was excluded
Replication	All attempts at replication were successful for at least twice on different time and institutes.
Randomization	For in vivo study, animal groups were randomized by body weight and/or age. For other experiments, all samples were randomly allocated into experimental groups.
Blinding	Experiments were all carried out blindingly.

Reporting for specific materials, systems and methods

We require information from authors about some types of materials, experimental systems and methods used in many studies. Here, indicate whether each material, system or method listed is relevant to your study. If you are not sure if a list item applies to your research, read the appropriate section before selecting a response.

Materials & experimental systems

n/a	Involved in the study	
☐	☒	Antibodies
☐	☒	Eukaryotic cell lines
☒	☐	Palaeontology and archaeology
☐	☒	Animals and other organisms
☒	☐	Human research participants
☒	☐	Clinical data
☒	☐	Dual use research of concern

Methods

n/a	Involved in the study	
☒	☐	ChIP-seq
☐	☒	Flow cytometry
☒	☐	MRI-based neuroimaging

Antibodies

Antibodies used	The antibodies used here were Brilliant Violet 605 anti-mouse CD45 (Biolegend #103140), APC anti-mouse CD326 (Biolegend #118214), APC/Cyanine7 anti-mouse CD31 (BioLegend #102440), PerCP-Cyanine 5.5 anti-mouse CD11b (BioLegend # 101228), and FITC anti-mouse CD11c (BioLegend #117306). The dilution ratio for all antibodies listed above was 1:200 with staining buffer (ThermoFisher #00422226).
Validation	All primary antibodies were bought from vendors (Biolegend), used for the specifies suggested by the manufacturers (mouse-specific). All antibodies were validated by the supplier. Validation statements are provided on the manufacturer's website. Each lot of this antibody is quality control tested by immunofluorescent staining with flow cytometric analysis.<br>(a) For Brilliant Violet 605 anti-mouse CD45 (Biolegend #103140), each lot of this antibody is quality control tested by immunofluorescent staining with flow cytometric analysis. For flow cytometric staining with µg, the suggested use of this reagent is ≤0.5 µg per million cells in 100 µl volume.<br>(b) For APC anti-mouse CD326(Biolegend #118214), each lot of this antibody is quality control tested by immunofluorescent staining with flow cytometric analysis. For flow cytometric staining, the suggested use of this reagent is ≤ 0.25 µg per 10^6 cells in 100 µl volume.<br>(c) For APC/Cyanine7 anti-mouse CD31 (BioLegend #102440), each lot of this antibody is quality control tested by immunofluorescent staining with flow cytometric analysis. For flow cytometric staining, the suggested use of this reagent is ≤ 0.5 µg per million cells in 100 µL volume.<br>(d) For PerCP-Cyanine 5.5 anti-mouse CD11b (BioLegend # 101228), each lot of this antibody is quality control tested by immunofluorescent staining with flow cytometric analysis. For flow cytometric staining, the suggested use of this reagent is = 0.25 µg per 106 cells in 100 µl volume.<br>(e) For FITC anti-mouse CD11c (BioLegend #117306), each lot of this antibody is quality control tested by immunofluorescent staining with flow cytometric analysis. For flow cytometric staining, the suggested use of this reagent is ≤0.25 µg per million cells in 100 µl volume.

Eukaryotic cell lines

Policy information about cell lines

Cell line source(s)	HepG2 cells, B16F10 purchased from ATCC were used.
Authentication	The cell lines were confirmed by morphology.
Mycoplasma contamination	Cell lines were tested negative for mycoplasma contamination.
Commonly misidentified lines (See ICLAC register)	No commonly misidentified cell lines were used in the study.

Animals and other organisms

Policy information about studies involving animals; ARRIVE guidelines recommended for reporting animal research

Laboratory animals	As reported in the Methods section, Female BALB/c mice (6 – 8 weeks) were obtained from the Jackson Laboratory and female Ai9 mice (6-8 weeks) bred in Johns Hopkins Animal Facilities.
Wild animals	The study did not involve wild animals.
Field-collected samples	The study did not involve samples collected from the field.
Ethics oversight	All animal procedures were performed with ethical compliance and approval by the Johns Hopkins Institutional Animal Care and Use Committee.

Note that full information on the approval of the study protocol must also be provided in the manuscript.

Flow Cytometry

Plots

Confirm that:

- [x] The axis labels state the marker and fluorochrome used (e.g. CD4-FITC).
- [x] The axis scales are clearly visible. Include numbers along axes only for bottom left plot of group (a 'group' is an analysis of identical markers).
- [x] All plots are contour plots with outliers or pseudocolor plots.
- [x] A numerical value for number of cells or percentage (with statistics) is provided.

Methodology

Sample preparation	As indicated in Method section, To quantify the mCherry+ or tdTom+ cells among different cell types in each organ, cell isolation and staining was performed, followed by flow cytometry analysis. For hepatocyte isolation, a two-step collagenase perfusion was executed as described previously. Briefly, mice were anesthetized using isoflurane then fixed. Perfusion, initially using liver perfusion medium (Thermo Fisher) for 7–10 min, then switching to liver digestion medium (Thermo Fisher) for another 7–10 min, was performed. The liver was collected on a plate containing 10 mL of liver digestion medium and cut to release the hepatocytes. The released hepatocytes were then collected and washed with ice-cold hepatocyte wash medium (Thermo Fisher) and centrifuged at 50g for 5 min. The supernatant was decanted, and the pellet was resuspended with an ice-cold hepatocyte wash medium. The cell suspension was passed through a 100-μm filter. The hepatocyte suspension was washed twice with ice-cold hepatocyte wash medium and once with PBS via centrifugation (50g) for 5 min. Afterwards, the hepatocytes were further strained through a 100-μm filter and centrifuged at 50g for 5 min, and cells were resuspended in 500 μL of staining buffer. The antibodies used here were Brilliant Violet 605 anti-mouse CD45 (Biolegend #103140), Cyanine 5 anti-mouse CD326 (Biolegend #118214), APC/Cyanine7 anti-mouse CD31 (BioLegend #102440), PerCP-Cyanine 5.5 anti-mouse CD11b (BioLegend # 101228), and FITC anti-mouse CD11c (BioLegend #117306). Flow data were acquired on SH800 and analyzed using FlowJo software.<br><br>For isolation and staining of spleen cells, the removed spleen was minced using a sterile blade and homogenized in 250 μL of digestion medium (45 units/μL collagenase I, 25 units/μL DNase I and 30 units/μL hyaluronidase). The spleen solution was transferred into a 15-mL tube that contained 5–10 mL of digestion medium. Next, the spleen solution was filtered using a 70-μm filter and washed once with PBS. Cells was pelleted at 300g for 5 min at 4 °C, and resuspended in 2 mL of red blood cell lysis buffer (BioLegend) and incubated on ice for 5 min. After incubation, 4 mL of cell staining buffer (BioLegend) was added and centrifuged again at 300 g for 5 min. Cell pellet was washed with staining buffer for 3 times and stained with antibodies (total volume 100 μL) for 20 min in the dark at 4 °C. The stained cells were washed twice with 1 mL of PBS, then resuspended in 500 μL PBS for flow cytometry analysis. The antibodies used include Brilliant Violet 605 anti-mouse CD45 (BioLegend), PerCP-Cyanine 5.5 anti-mouse CD11b (BioLegend), APC anti-mouse CD11c, FITC anti-mouse CD3 and PE-Cyanine 7 anti-mouse CD19 (BioLegend).<br><br>For isolation and staining of lung cells, isolated lungs were minced using a sterile blade and then transferred into a 15-mL tube that contained 10 mL of 2× digestion medium (90 units/μL collagenase I, 50 units/μL DNase I, and 60 units/μL hyaluronidase) and incubated at 37 °C for 1 h with shaking. After incubation, any remaining lung tissue was homogenized.

	The following steps were similar to the spleen protocol described above. The antibodies used here were the same to that of hepatocytes.
Instrument	Sony SH800S cell sorter
Software	Collection: SH800S and Analysis: FlowJo.
Cell population abundance	Sorting was not done. But for FACS gating analysis: cell populations of interest (ie., hepatocytes) were abundant (collected as much as possible from the samples ~ 1000,00, based on sample source)
Gating strategy	Gating strategies are referred to those described in the BioLegend website. Gating was first based on FSC/SSC together with FSC-A/FSC-H (singlet populations). The cell populations within the gate were further analyzed based on expression of targetd protein. Single positive staining were used to determine the positive.

☒ Tick this box to confirm that a figure exemplifying the gating strategy is provided in the Supplementary Information.
